# *FLI1* enhances the malignant phenotype of glioma cells and exerts immunomodulatory effects through feedback crosstalk with exonic circRNA *FECR1* and interferon-induced *ISG15*

**DOI:** 10.3892/ijmm.2026.5912

**Published:** 2026-07-01

**Authors:** Xue Wen, Xiaoyi Gu, Xu Yan, Naifei Chen, Lei Zhou, Hui Li, Andrew R. Hoffman, Wei Li, Ji-Fan Hu, Jiuwei Cui

**Affiliations:** 1 Cancer Center, First Hospital of Jilin University, Changchun, Jilin 130021, P.R. China; 2 Department of Medicine, Stanford University School of Medicine, Stanford, CA 94305, USA

**Keywords:** FLI-1, ISG15, glioma, epigenetics, immunoregulatory, feedback circuitry

## Abstract

Gliomas comprise a group of common primary brain tumors with a high degree of malignancy and a poor prognosis. There are currently no targeted therapeutics for glioma in clinical practice. The present study revealed a novel crosstalk circuitry mechanism that contributes to the overexpression of Friend leukemia virus integration 1 (*FLI1*) in glioma. *FLI1*, a member of the ETS transcription factor family, was upregulated in glioma and its expression was associated with malignant phenotype and poor prognosis of the disease. Notably, aberrant *FLI1* expression in glioma was regulated by its exonic circRNA *FECR1* through a positive feedback mechanism. *FLI1* knockdown suppresses tumor phenotypes of glioma cells. Using RNA-seq and Co-IP assays, the present study identified interferon-stimulated gene 15 (*ISG15*), a paracrine factor known to reprogram the tumor immunosuppressive microenvironment, as a new molecular target of *FLI1* in glioma. *FLI1* coordinated with *ISG15* to suppress immune function, including the secretion of the cytokines perforin, IFN-γ, TNF-α and IL2 from T cells, as well as perforin from γδ T cells. Mechanistically, *FLI1* bound to the *ISG15* promoter regulatory elements, where it activates the *ISG15* gene by orchestrating an active intrachromosomal spatial loop with characteristic DNA hypomethylation and histone H3K9 and H3K27 acetylation. As a downstream target, ISG15 also participated in a positive feedback loop with FLI1 by enhancing its stability from ubiquitination-induced degradation. Thus, targeting this *FLI1*-*ISG15* feedback circuitry may provide a novel strategy to develop therapeutics for gliomas.

## Introduction

Gliomas are a type of primary neurological tumor of the brain. Among these tumors, glioblastoma is classified as a grade IV glioma by the World Health Organization and is the most malignant type, with a median overall survival of ~15 months and a 5-year survival rate of <10% despite aggressive treatment (1,2). Surgical resection, chemotherapy and radiotherapy are currently the established treatments (3). However, glioblastomas reside in a highly immunosuppressed microenvironment and are therefore susceptible to recurrence and metastasis (1). Since there are currently no effective targeted therapeutics for glioblastoma, there is an urgent need to find new molecular targets to improve therapeutic efficacy and prolong the survival of patients with glioblastoma.

Friend leukemia virus integration 1 (*FLI1*) is a member of the *E26* transformation-specific transcription factor family (4). *FLI1* plays an important role in regulating the self-renewal and differentiation of hematopoietic stem cells (5). Abnormally high *FLI1* expression can induce the development of erythroleukemia and play a key driving role in hematological tumor development (6,7). Previous studies have reported that in addition to hematopoietic malignancies, *FLI1* is also abnormally expressed in a variety of solid tumors, including Ewing's sarcoma (8), small cell lung cancer (SCLC) (9) and breast cancer (10,11). In highly invasive SCLC cell lines, *FLI1* knockdown can promote apoptosis, induce inhibition of cell proliferation and increase tumor colony formation and tumorigenicity *in vivo* (12).

*FLI1* also plays a key role in modulating the tumor immune microenvironment, which has a profound impact on tumor growth, invasion, metastasis and treatment response. For example, *FLI1* is involved in immune regulation in breast cancer (13). CXCL13, a chemokine derived from B cells and regulatory T cells, is upregulated in macrophages due to *FLI1* deficiency, leading to immune activation in tissue fibrosis, vasculopathy and systemic sclerosis (14). *FLI1* depletion increases the proportion of Th2- and Th17-like Tregs in bleomycin-induced skin fibrosis (15). Its potential as an immune target has previously been examined (13-15). These findings suggest that *FLI1* may prove to be a potential therapeutic target for a variety of malignant neoplasms. However, the specific role of *FLI1* in the development of gliomas, including its immunomodulatory function, has not been examined.

However, the specific role of *FLI1* in glioma progression remains unclear. The present study aimed to elucidate the upstream regulatory circuitry of *FLI1* and identify its key downstream effectors, with a specific focus on Interferon-stimulated gene 15 (*ISG15*). As a pivotal type I IFN-stimulated paracrine factor, *ISG15* plays a critical role in modulating the tumor microenvironment (16-19). By integrating RNA sequencing (RNA-seq), co-immunoprecipitation (Co-IP), and comprehensive molecular assays, the present study characterized the epigenetic mechanisms by which *FLI1* regulates *ISG15*. Furthermore, the potential feedback loops between *FLI1* and *ISG15* as well as their crosstalk on the regulation of T cell-mediated immune responses were investigated, thus providing a theoretical basis for novel glioma therapeutics.

## Materials and methods

### Cell lines, antibodies and reagents

The human glioblastoma (U251MG) cell lines and viral packaging 293T cells were obtained from the Shanghai Institute of Cell Biology at the Chinese Academy of Sciences. The human glioblastoma cell line U87MG, obtained from Beijing Solarbio Science & Technology Co., Ltd. (cat. no. SCC-112411), is an ATCC version, which is derived from a glioblastoma of unknown origin. The identity of the cell line was confirmed by short tandem repeat profiling. All cells were cultured in DMEM) supplemented with 10% fetal bovine serum (Gibco; Thermo Fisher Scientific, Inc.), 100 U/ml streptomycin and 100 U/ml penicillin (Hanbio Biotechnology Co., Ltd.) at 37°C with 5% CO_2_ in a humidified atmosphere.

The following antibodies were used: FLI1 (cat. no. ab133485, Abcam; cat. no. MA1-196; Thermo Fisher Scientific, Inc.), ISG15 (cat. no. ab285367; Abcam), PI3 Kinase (C73F8) (cat. no. 4249S; Cell Signaling Technology Inc.), Akt (cat. no. 4691S; Cell Signaling Technology Inc.) and Akt phosphorylated on serine 473 (phospho-Akt [Ser473]) (cat. no. 4060S, Cell Signaling Technology Inc.), phospho-c-Raf (Ser259) (cat. no. 9421S; Cell Signaling Technology Inc.), MEK1 (cat. no. SC-6250; Santa Cruz Biotechnology, Inc.), phospho-MEK1/2 (Ser217/221) (cat. no. 2926S, Cell Signaling Technology Inc.), ERK1 (cat. no. SC-94, Santa Cruz Biotechnology, Inc.), p-ERK1/2 (cat. no. 9106S; Cell Signaling Technology Inc.), P38MAPK (D13E1) (cat. no. 4511S; Cell Signaling Technology Inc.), P-P38 (Thr180/tyr182) (cat. no. 4937s; Cell Signaling Technology Inc.), Caspase 8 (Asp391) (cat. no. 9496S; Cell Signaling Technology Inc.), P27 (cat. no. SC-528; Santa Cruz Biotechnology, Inc.), β-Actin (cat. no. AA128; Beyotime Biotechnology), HRP-labeled goat anti-mouse IgG (H+L) (cat. no. A0216; Beyotime Biotechnology) and HRP-labeled goat anti-rabbit IgG (H+L) (cat. no. A0208; Beyotime Biotechnology).

The JetPRIME transfection reagent (Polyplus-transfection SA) was used for transient transfection of DNA and short interfering RNA (siRNA) into glioblastoma cells, respectively. The siRNA sequences are shown in Table SI. To knockdown the target gene, the present study employed a siRNA pool strategy by transfecting a mixture of multiple siRNAs targeting the same gene, rather than using individual siRNAs. The western blotting data confirmed that the siRNA pool effectively silenced the target gene in the experimental setting. Cells for Transwell assays were seeded into the upper chamber of a 24-well-transwell unit with 8 m pores (cat. no. 3422, Corning, Inc.) coated with Matrigel (cat. no. 356234; BD Biosciences).

### Construction of plasmids and viral transfection

Two short hairpin RNAs (shRNA) against *FLI1* or *ISG15* mRNA were inserted into the lentiviral vector (plasmid no. 8453; Addgene, Inc.). The shRNA sequences are shown in Table SII. Two single-guide RNAs (sgRNA; FLL1-1: GCC TCG GGG AGT CCT GAC TA; FLL1-2: CAG TGA GGG TCA ACG TCA AG), designed to target exon 2, were inserted into the lenti CRISPR V2 vector (plasmid no. 52961; Addgene, Inc.). The plasmid overexpressing the full length *FLI1* coding sequence was constructed into the pLVX-Puro-EGFP-FLAG vector. After confirmation by DNA sequencing, the lentiviruses were packaged in 293 cells using Jet PRIME transfection reagent (cat. no. 101000046; Polyplus). For lentivirus packaging, 293 cells were seeded in 6-well plates and transfected with 1 *µ*g of each lentiviral plasmid and packaging plasmids following the protocol provided by the manufacturer. The virus-containing supernatants were collected 48 and 72 h post-transfection, and concentrated with Centrifugal Filter Units (Amicon Ultra-15; MilliporeSigma). Glioblastoma cells in six-well plates were infected with lentiviruses using polybrene (8 ug/ml). At 3 days post-infection, glioblastoma cells were selected by puromycin. After puromycin selection, mixed stable cells were collected for each group and were used for gene analysis by reverse transcription-quantitative PCR (RT-qPCR) and western blot analysis.

### RNA isolation and RT-qPCR quantitation

U87MG and U251MG cells were washed twice with cold PBS, detached using 0.25% trypsin-EDTA and collected by centrifugation at 800 × g for 5 min at 4°C. Cells (1×10^7^ cells per group) were immediately lysed using TRI reagent^®^ (cat. no. T9424; MilliporeSigma). The extracted total RNA was stored at -80°C. cDNA was synthesized using RNA reverse transcriptase (Shanghai Yeasen Biotechnology Co., Ltd.) and target amplification was performed with a Bio-Rad Thermol Cycler. PCR of 1 cycle at 95°C for 2 min, 32 cycles at 95°C for 15 sec, 60°C for 15 sec and 72°C for 15 sec and 1 cycle at 72°C for 10 min. qPCR was performed using SYBR GREEN PCR Master (Shanghai Yeasen Biotechnology Co., Ltd.); the Cq values of target genes were assessed by qPCR in triplicate using a sequence detector (Bio-Rad 384; Bio-Rad Laboratories, Inc.) and were normalized over the Cq of the β-Actin control (20). Primers used for PCR quantitation are listed in Table SIII.

### Glioma tissue samples

The study was approved by the Research Ethics Committee of the First Hospital of Jilin University (Changchun, China; approval no. 2024-1276). A waiver of informed consent was obtained from the ethics committee as the formalin-fixed and paraffin-embedded tissues of glioma samples were obtained from the biological sample library of the First Hospital of Jilin University (Changchun, China). The pathological diagnosis was made in accordance with the histological classification of tumors developed by the World Health Organization. Inclusion criteria were: Patients with histologically or cytologically confirmed glioma; age ≥18 years, regardless of sex and a BMI between 19 and 28 kg/m^2^ (inclusive). Exclusion criteria were: Patients with uncontrolled infections; patients positive for HIV, active hepatitis B (HBsAg-positive and HBV-DNA-positive) or hepatitis C (HCV antibody-positive); and those who had participated in other drug clinical trials.

### Immunohistochemical staining

Expression of genes in tumor samples was evaluated by immunohistochemical staining as previously described (21). Briefly, tissue slides were deparaffinized, rehydrated, and incubated in 3% hydrogen peroxide for 15 min at room temperature to block endogenous peroxidase activity. The slides were incubated with primary polyclonal antibodies, including rabbit anti-FLI1 monoclonal antibody (1:140; cat. no. ab133485; Abcam) and rabbit anti-ISG15 monoclonal antibody (1:500; cat. no. ab285367; Abcam) at 4°C overnight. After washing with PBS, the slides were incubated with an HRP-conjugated goat anti-rabbit IgG (H+L) secondary antibody (1:5,000; cat. no. P0948; Beyotime Biotechnology) at room temperature for 1 h. The *FLI1* and *ISG15* signals were visualized using a HRP DAB Detection kit. The abundance of *FLI1* and *ISG15* was quantitated by immunoreactivity scoring as evaluated by two independent investigators. The pathological diagnosis was based on the 5th edition of World Health Organization (WHO) Central Nervous System (CNS) tumor classification (2).

### Western blot analysis

U87MG and U251MG cells were lysed in the RIPA lysis buffer (cat. no. P0013B; Beyotime Biotechnology) freshly supplemented with the protease inhibitor cocktail and 1 mM PMSF on ice. The lysates were centrifuged at 12,000 × g for 15 min at 4°C, and the supernatants were collected. Protein concentration was determined using the BCA Protein Assay Kit (cat. no. P0012; Beyotime Biotechnology). The samples were then mixed with 1 × SDS-PAGE loading buffer and boiled at 95°C for 10 min. A total of 30 *µ*g of protein per sample was resolved by 10% SDS-PAGE and then transferred to a PVDF membrane. The PVDF membranes were blocked with Blocking Buffer (cat. no. P0023B; Beyotime Biotechnology) at room temperature for 1 h, followed by incubation with primary antibodies (FLI1, ISG15, PI3K, Akt, MEK1, ERK1 and p38 MAPK at 1:1,000; Flag and β-Actin at 1:3,000) overnight at 4°C. After washing 3 times with TBS-Tween (TBS containing 0.1% Tween-20; TBST), the membranes were incubated with HRP-labeled goat anti-mouse IgG (H+L) (1:3,000; cat. no. A0216; Beyotime Biotechnology) or HRP-labeled goat anti-rabbit IgG (H+L) (1:3,000; cat. no. A0208; Beyotime Biotechnology) at room temperature for 2 h. Signals were visualized using SuperSignal West Pico PLUS (cat. no. 34580; Thermo Fisher Scientific Inc.) and images were captured using a gel imaging system. Due to limited lanes in Western gel, total and phosphorylated proteins, including MEK and p-MEK, were measured on separate gels. To ensure comparability, all the blots for total protein and phosphorylated protein were run concurrently on separate gels using the same amount of protein and identical electrophoresis/western blotting conditions. Moreover, the sample order on each gel is strictly aligned (one-to-one correspondence). For each experimental run, the present study included loading controls (β-Actin). The quantification and normalization were performed strictly within each individual blot. However, despite extensive optimization, the total C-RAF antibody consistently produced non-specific signals, preventing a clear and interpretable result from being obtained. As a result, only the data for p-C-RAF are shown. For the p-C-RAF assay, all blots presented (p-C-RAF and loading control) were derived from the same experimental run.

### Integration of RNA-Sequencing (RNA-seq) and binding proteins data

To identify FLI1-associated target molecules, total RNA was extracted from FLI1-knockdown (shFLI1) and control (pGreen) cells using TRI reagent^®^ (cat. no. T9424; MilliporeSigma). The quality and integrity of the RNA were assessed using an Agilent 2100 Bioanalyzer (Agilent Technologies, Inc.) with an RNA 6000 Nano Kit. Only RNA samples with an RNA Integrity Number (RIN) ≥7.0 were used for subsequent library construction. Sequencing libraries were constructed by BGI Genomics using the MGIEasy RNA Library Prep Kit. Briefly, mRNA was purified from total RNA using poly-T oligo-attached magnetic beads. Fragmentation was carried out using divalent cations under elevated temperature. First-strand cDNA was synthesized using random hexamer primers, followed by second-strand cDNA synthesis. The library fragments were purified, end-repaired, A-tailed and ligated with adapters. The products were then purified and enriched by PCR to create the final cDNA library. The library quality was assessed using the Agilent Bioanalyzer 2100 system. The libraries were sequenced on the DNBSEQ-T7 platform (MGI Tech Co., Ltd.) by BGI Group. The sequencing was performed using the DNBSEQ-T7 RS Reagent Kit (cat. no. 1000020036; MGI Tech Co., Ltd.) to generate 150 bp paired-end reads. The final library loading concentration was 100-200 pM, quantified by Qubit dsDNA HS Assay Kit and qPCR. Raw sequencing reads were processed to remove adapters and low-quality reads. Clean reads were aligned to the human reference genome GRCh38/hg38 using HISAT2 (v2.1.0; http://ccb.jhu.edu/software/hisat2). Gene expression levels were calculated as Fragments Per Kilobase of transcript per Million mapped reads (FPKM) using StringTie (v1.3.4d; https://ccb.jhu.edu/software/stringtie). Differentially expressed genes between shFLI1 and pGreen groups were identified using the DESeq2(v1.34.0; https://bioconductor.org/packages/release/bioc/html/DESeq2.html) R package. Differentially expressed RNAs were calculated as the log2-transformed (shFLI1/pGreen) gene expression values (Fold Change). The Kyoto Encyclopedia of Genes and Genomes (22) (KEGG; https://www.genome.jp/kegg/) pathway analysis (KEGG_PATHWAY) were performed by using HIPLOT (https://hiplot.com.cn; accessed on 22 March 2024). FLI1-binding proteins were mapped by FLI1 pull-down mass spectrometry sequencing in U87MG and U251MG (Shanghai Zhongke New Life Biotechnology Co., Ltd.). IgG was used as the control. The target molecules were merged with the RNA-seq and FLI1-binding proteins data using the VENN program (https://jvenn.toulouse.inrae.fr/app/example.html). Venn diagrams were used to visualize the overlap molecules between the datasets. The overlapping molecules identified by the datasets were chosen for further functional characterization.

### RNA FISH

RNA *in situ* hybridization was performed using specific fluorescent probes targeting the back-splice junction of exonic circRNA FECR1 (Probe sequence: 5′-Cy3-GAC AGA GCC UCU UUC CCU GAG G-3′; Guangzhou RiboBio Co., Ltd.). U87MG and U251MG cells were grown to the exponential phase and were 50 to 60% confluent at the time of fixation in 4% paraformaldehyde at room temperature for 10 min. Subsequently, permeabilization was performed using 0.5% Triton X100 for 10 min at 4°C. After prehybridization (1xPBS/0.5% Triton X-100), cells were hybridized in hybridization buffer with 20 nmol/l probes specific to FECRs at 37°C overnight. For nuclear counterstaining, cells were incubated with DAPI for 10 min. The images were acquired on a FV3000 confocal microscope (Olympus Corporation).

### Chromatin RNA in situ reverse transcription (CRIST) assay

A CRIST assay (23,24) was modified to identify *FECR1* binding to *FLI1*. Specifically, cells were cross-linked with 2% formaldehyde and lysed with hypotonic buffer (10 mM Hepes, pH 7.9, 1.5 mM MgCl2, 10 mM KCl, 0.4% NP-40, RNase inhibitor 100 U/ml, 1x protease inhibitors). Nuclei were suspended in 1x reverse transcription buffer. RT was performed using *FECR1*-specific antisense primers (AGT CCC TTT CTC CGA GAC AGC C) and biotin-dCTP. To reduce non-specific reaction, the transcription was performed with Maxima Reverse Transcriptase (Thermo Fisher Scientific, Inc.) at 65°C for 50 min. The reaction was stopped by adding 4 *µ*l 0.5 M EDTA. After nuclear lysis, the chromatin complex was subjected to sonication on ice. The biotin-FECR1 cDNA/chromatin DNA complex was pulled down with biotin-streptavidin magic beads (Invitrogen; Thermo Fisher Scientific, Inc.). After reversing the cross-links and washing with 10 mg/ml proteinase K at 65°C for 2 h and treatment with 0.4 *µ*g/ml RNase A at 37°C for 30 min, the genomic DNA that interacts with the circular RNA was extracted and analyzed by PCR and agarose gel electrophoresis. Primers used for PCR are listed in Table SIV.

### Coimmunoprecipitation (co-IP)

U87MG and U251MG cells were subjected to lysis at 4°C using a lysis buffer containing 25 mM Tris-HCl, pH 7.5, 1% NP-40, 150 mM NaCl, 1 mM EDTA and 5% glycerin. Following centrifugation (12,000 × g; 4°C; 20 min), the supernatant underwent overnight immunoprecipitation at 4°C using anti-Flag antibody (cat. no. F3165; MilliporeSigma; 5 *µ*g), anti-FLI1 antibody (5 *µ*g; cat. no. ab133485; Abcam), anti-ISG15 antibody (5 *µ*g; cat. no. ab285367; Abcam), and normal IgG (5 *µ*g; cat. no. ab172730; Abcam). Next, the lysate (500 *µ*g of protein per IP reaction) was incubated with Pierce protein A/G Magnetic Beads (cat. no. 88802, Thermo Fisher Scientific, Inc.), the immunoprecipitated complexes were isolated using a magnetic separator and washed 5 times with lysis buffer containing 25 mM Tris-HCl, pH 7.5, 150 mM NaCl, 1 mM EDTA and 5% glycerin. The complexes were eluted by adding 1x SDS-PAGE loading buffer and boiling at 95°C for 10 min. The eluates were then evaluated via western blotting.

### Immunofluorescence

U87MG or U251MG cells were grown to the exponential phase and were 50 to 60% confluent at the time of fixation in 4% paraformaldehyde at room temperature for 10 min. Subsequently, permeabilization was performed using 0.5% Triton X100 for 10 min at 4°C. Finally, the cells were dried on slides. After these steps, the cells were treated with 1% BSA in PBS for blocking at room temperature for 1 h and subsequently incubated with antibodies against ISG15 (cat. no. ab285367; Abcam) diluted 1:100 in PBS. Following overnight incubation at 4°C, the cells were incubated with fluorescent secondary antibodies conjugated with Alexa Fluor 555 (cat. no. A-31570; Invitrogen; Thermo Fisher Scientific, Inc.) for 1 h at room temperature. The cells were then stained with DAPI at room temperature for 10 min, washed with PBS and fixed on slides. Images were acquired with an FV3000 confocal microscope (Olympus Corporation).

### Molecular docking

We performed molecular docking using HADDOCK to predict the binding interface between FLI1 and ISG15. The acquisition of protein structures was obtained by downloading full-length three-dimensional structure files from the UniProt database (https://www.uniprot.org/). Next, the prediction of interacting amino acid residues resulted from protein-protein docking simulations that were performed using HADDOCK. The resulting complex models were submitted to PDBePISA (https://www.ebi.ac.uk/pdbe/pisa/) to identify specific interfacial amino acid residues and to calculate the binding energy. Finally, the docking results were visualized and rendered as 3D structural models using PyMOL software, version 2.5.10 (Schrödinger, LLC).

### Isolation and culture of human primary activated T cells and γδ T cells

Both human primary activated T cells and γδ T cells were isolated from commercial peripheral blood mononuclear cells (PBMCs) purchased from Shunlitong Pharmaceutical Technology Co., a subsidiary of Miaoshun (Shanghai) Biotechnology Co., Ltd. The cells were approved by the Research Ethics Committee with the approval no. 2023-12. Human primary T cells (CD3+) were purified from human PBMCs using the MagniSort™ Human CD3 Positive Selection Kit (cat. no. 8802-6830-74; Thermo Fisher Scientific, Inc.) according to the manufacturer's instructions. The sorted cells were cultured in T-cell specific medium and stimulated for activation and proliferation using anti-CD3/anti-CD28 antibody-conjugated magnetic beads and recombinant human IL-2 (300 IU/ml). For γδ T cell expansion, PBMCs isolated from healthy donors served as the starting material. Cells were stimulated with zoledronic acid combined with recombinant human IL-2 (500-1,000 IU/ml) to induce selective activation and expansion of γδ T cells.

### Flow cytometry

For surface staining, both human primary activated T cells and human primary activated γδ T cells were incubated with APC-anti-human CD3+ antibody (cat. no. 372706; BioLegend, Inc.), FITC-anti-human Vγ9 TCR antibody (cat. no. 555732; BD Biosciences) and FITC-anti-human CD56 antibody (cat. no. 304604; BioLegend, Inc.), at 25°C for 20 min in 1x PBS buffer. For intracellular staining, cytokine staining was performed on human primary activated T cells and human primary activated γδ T cells utilizing the BD Cytofix/Cytoperm Fixation/Permeabilization Solution Kit with BD GolgiPlug (cat. no. 555028; BD Biosciences) with PE-anti-human Granzyme B antibody (cat. no. 561142; BD Biosciences), PE-anti-human Perforin antibody (cat. no. 353304; BD Biosciences), PE-anti-human IFN-γ antibody (cat. no. 502509; BD Biosciences) PE-anti-human IL-2 antibody (cat. no. 554566; BD Biosciences) and PE-anti-human TNF-α antibody (cat. no. 502909; BD Biosciences). Following staining, the cells were washed with buffer before being analyzed with a BD FACS Aria II flow cytometer (BD Biosciences). The data were analyzed utilizing version 10 of FlowJo software (Tree Star, Inc.).

### Chromatin immunoprecipitation (ChIP) assay

ChIP assay was used to identify *FLI1* binding in the *ISG15* promoter and to quantify histone modifications as previously described (25). Briefly, 1.0×10^7^ cells were fixed with 2% formaldehyde at room temperature for 10 min and lysed with hypotonic buffer (10 mM Hepes, pH 7.9, 1.5 mM MgCl2, 10 mM KCl, contain 0.4% NP-40, supplemented with protease inhibitors). Nuclei were collected, suspended in sonication buffer (50 mM Tris-HCL pH 7.4, 2.5 mM EDTA, 0.5% SDS, supplemented with protease inhibitors) and then sonicated for 180 sec (10 sec on and 15 sec off) on ice with a sonicator with a 2-mm microtip at 33% output control. The sonicated chromatin was collected by centrifugation (12,000 × g; 4°C; 20 min), aliquoted and stored at -80°C. Protein A/G Magnetic Beads and a specific anti-FLI1 (cat. no. MA1-196; Thermo Fisher Scientific Inc.), anti-Acetyl-histone H3 (Lys9) and anti-Acetyl-histone H3 (Lys27) antibody (Cell Signaling Technology Inc.) were incubated with rotation for 30 min at room temperature. The sonication supernatant and beads were incubated with antibody at 4°C on a rotating rack overnight. For the ChIP assay, *BCL2*, a key gene controlled by *FLI1*, was used as the positive control in the *FLI1* binding assay. Anti-IgG was used as the ChIP control in parallel with testing samples. Precipitated DNA was subjected to qPCR and expressed as fold-enrichment compared to the IgG chromatin input. Human primers used for ChIP assay are listed in Table SV.

### Chromosome conformation capture (3C)

3C assays were performed to determine long-range intrachromosomal interactions as previously described (26). Briefly, target cells were cultured to 90% confluence (1.0×10^7^), cells were cross-linked with 2% formaldehyde at room temperature for 10 min and lysed with hypotonic buffer (10 mM Hepes, pH 7.9, 1.5 mM MgCl2, 10 mM KCl, contain 0.4% NP-40, supplemented with protease inhibitors). Nuclei were collected and suspended in 1x restriction enzyme buffer. An aliquot of nuclei (1×10^6^) was digested with 300U of restriction enzyme *MboI* at 37°C overnight. After stopping the reaction by adding SDS and incubating the mixture at 65°C for 20 min, chromatin DNA was diluted with NEB ligation reaction buffer; 2 *µ*g DNA was ligated with 2000U of T4 DNA ligase (New England BioLabs, Inc.) at 16°C for 4 h and then at room temperature for 30 min (final DNA concentration, 2.5 *µ*g/ml). After treatment with 10 mg/ml proteinase K at 65°C for 4-16 h or overnight to reverse cross-links and with 0.4 *µ*g/ml RNase A for 30 min at 37°C, DNA was extracted with dBIOZOL Genomic DNA Extraction Reagent (BSC16M1; Hangzhou Borui Technology Co., Ltd.) and detected by PCR amplification of the ligated DNA products. 3C PCR products were cloned and sequenced to validate the intrachromosomal interactions by assessing the presence of the *MboI* ligation site. The 3C interactions were quantitated by qPCR and standardized over the 3C ligation control. For comparison, the relative 3C interaction was calculated by setting the control as 1. Human primers used for 3C assay are listed in Table SVI.

### DNA methylation analysis

Genomic DNA was collected from cells using dBIOZOL Genomic DNA Extraction Reagent (BSC16M1; Hangzhou Borui Technology Co., Ltd.) following the manufacturer's instructions. DNA was treated with EZ DNA Methylation-Gold™ Kit (Zymo Research Corp.), and PCR was performed using DNA methylation-specific primers designed for the promoter of *ISG15* site (Table SVII). To examine the status of DNA methylation at every CpG site, the amplified PCR DNAs were cloned into pJET1.2/blunt cloning vector (cat. no. K1231; Thermo Fisher Scientific, Inc.) and transformed into Trans10 Chemically Competent Cell. Plasmid DNA was collected by Wizard^®^ Plasmid DNA Purification kit (cat. no. A1223; Promega Corporation) and sequenced.

### Statistical analysis

The experimental data are presented as mean ± SD and were derived from at least three biological replicates. Statistical analyses were performed using GraphPad Prism v7.0 (GraphPad Software; Dotmatics). Unpaired two tailed Student's t-tests or one-way ANOVA (Bonferroni post hoc test) were used for comparison among groups. The level of significance was indicated as ^*^P<0.05, ^**^P<0.01 and ^***^P<0.001, unless stated otherwise.

## Results

### FLI1 correlates with a malignant phenotype and poor prognosis of glioma

**Figure 1:**
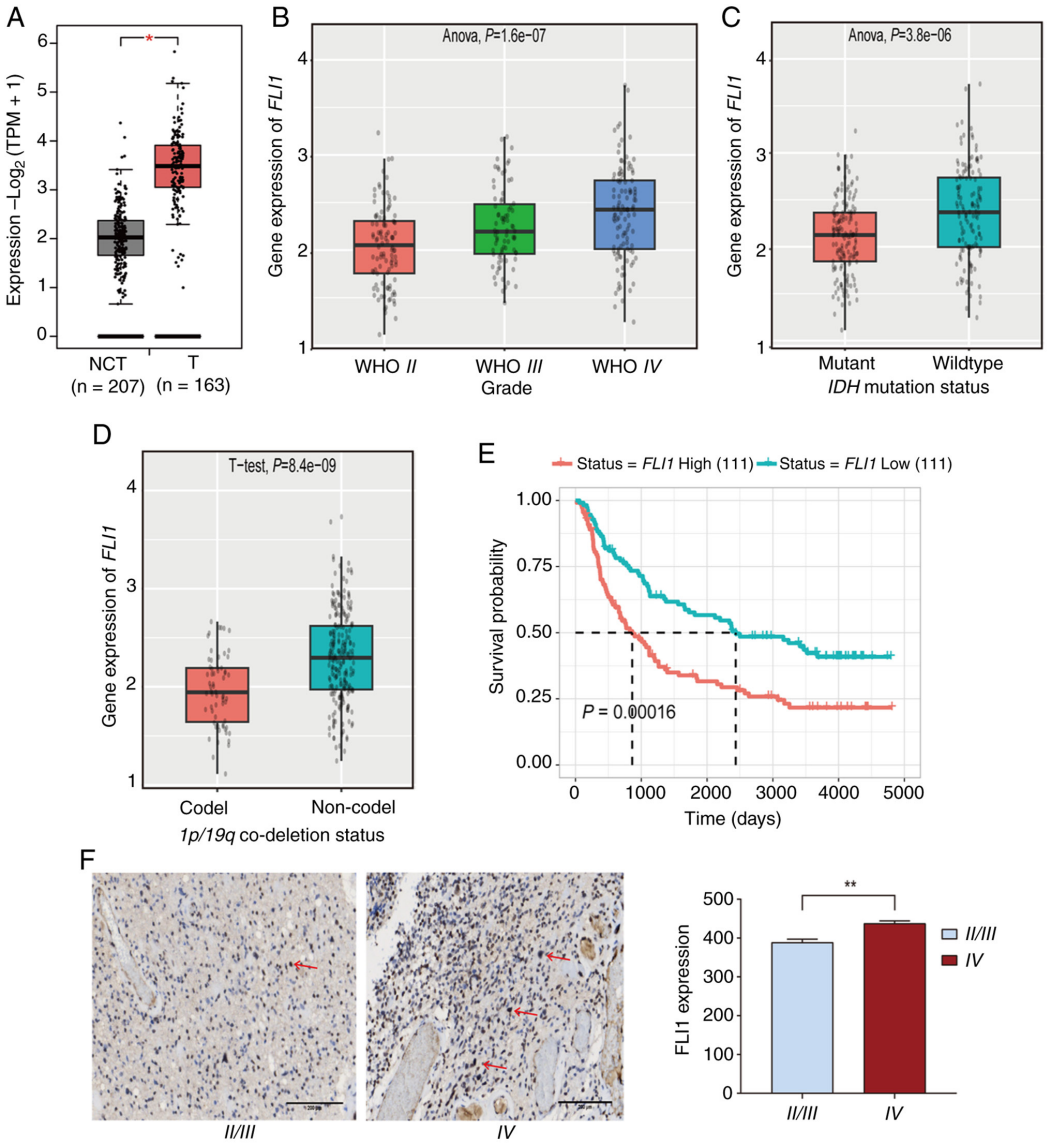
*FLI1* is associated with the development of glioma. (A) Upregulation of *FLI1* in glioma. *FLI1* was highly expressed in glioma as compared with normal brain specimens (P<0.05). (B) Histological grade of glioma. The expression level of *FLI1* was positively associated with the histological grade of gliomas. One-way ANOVA demonstrated that *FLI1* levels varied significantly among the different histological grades (P=1.6x^−07^). (C) *FLI1* expression in *IDH* mutation and *IDH* wild-type cells. One-way ANOVA showed a highly significant difference in *FLI1* levels between the two groups (P=3.8x^−06^). (D) *FLI1* expression in *1p/19q* co-deletion and Non-codel cells. *FLI1* levels were significantly lower in the *1p/19q* co-deletion group compared with the Non-codel group (P=8.4x^−09^). (E) Overall survival. Elevated *FLI1* expression was associated with reduced overall survival (P=0.00016). (F) Overexpression of *FLI1* in glioma tissues. *FLI1* expression was quantitated by immunohistochemical staining and was quantitated as an expression score. Red arrow, dark brown immunohistochemical staining of *FLI1* oncoprotein. ^**^P<0.01 in *IV* GBMs tissues as compared with *II/III* glioma tissues. Scale bar, 200 *µ*m. The gene expression data in Fig. 1A were generated from the GEPIA web server http://gepia.cancer-pku.cn/index.html. Histological grade, molecular features and survival data in Fig. 1B-E were retrieved from Chinese Glioma Genome Atlas https://www.cgga.org.cn/index.jsp. NTC, non-tumor controls; T, tumors; n, sample numbers; *IDH*, isocitrate dehydrogenase.

*FLI1* is an oncogenic driver in hematological malignancies, but its role in solid tumors remains to be characterized. To determine the role of *FLI1* in glioma, the present study first analyzed the expression of *FLI1* in tumor samples from patients with glioma using the TCGA and GEPIA databases. Analysis revealed that *FLI1* was significantly upregulated in glioma tumor tissues (n=163) as compared with normal tissues (n=207) (Fig. 1A). Moreover, *FLI1* was positively associated with the histological grade of glioma (Fig. 1B). The TCGA and GEPIA databases did not have the treatment data available for stratified survival analyses. Thus, the present study used its available data to perform the comparative analyses for IDH mutation and 1p/19q co-deletion molecular subtypes. Analysis revealed that *FLI1* expression levels inversely correlated with glioma prognosis at the molecular subtype level. *FLI1* expression was significantly higher in poor-prognosis glioblastomas (*IDH*-wildtype, *1p/19q* non-co-deletion) when compared with that in favorable-prognosis oligodendrogliomas (*IDH*-mutant, *1p/19q* co-deletion; Fig. 1C and D). The abnormally high expression of *FLI1* was associated with a malignant phenotype and poor prognosis of glioma (Fig. 1E). Furthermore, immunohistochemical staining of *FLI1* oncoprotein showed the most abundant FLI1 in grade IV glioblastoma (Fig. 1F; red arrows).

### Aberrant FLI1 expression in glioblastoma cell line is regulated by its exonic circRNA FECR1 through a positive feedback mechanism

Using a chromatin RNA *in situ* reverse transcription sequencing (CRIST-seq) approach (23,24), we previously identified the *FLI1* exonic circRNA *FECR1* as a positive feedback regulator that activates *FLI1* in breast cancer using a cis epigenetic mechanism (11). The present study thus explored whether this regulatory mechanism was also involved in the aberrant expression of *FLI1* in glioblastoma cells (Fig. S1A). Analysis revealed that exonic circRNA *FECR1* was expressed in U87MG cells. Sequencing showed that exonic circRNA *FECR1* was generated by reverse splicing of *FLI1* exons 4-2-3 (Fig. S1B). Fluorescence *in situ* hybridization showed that exonic circRNA *FECR1* was present in both the nucleus and the cytoplasm (Fig. S1C).

Using an RNA reverse transcription-associated trap (RAT) assay, the present study found that exonic circRNA *FECR1* interacted with the *FLI1* promoter (Fig. S2A, P1 and P2 sites), where it upregulated the gene through a positive feedback mechanism (23,24). Using gain- and loss-of-function assays, the present study found that exonic circRNA *FECR1* altered *FLI1* expression. After exonic circRNA *FECR1* overexpression (Fig. S2B, left panel), FLI1 protein was significantly upregulated as quantitated by western blotting (Fig. S2B, right panel). By contrast, exonic circRNA *FECR1* knockdown (Fig. S2C, left panel) significantly reduced FLI1 protein levels in glioblastoma cells (Fig. S2C, right panel). Thus, *FLI1* was upregulated by its exonic circRNA *FECR1* through a positive feedback mechanism in glioblastoma cells.

### Knockdown of FLI1 suppresses the tumor phenotypes of glio-blastoma cells

**Figure 2:**
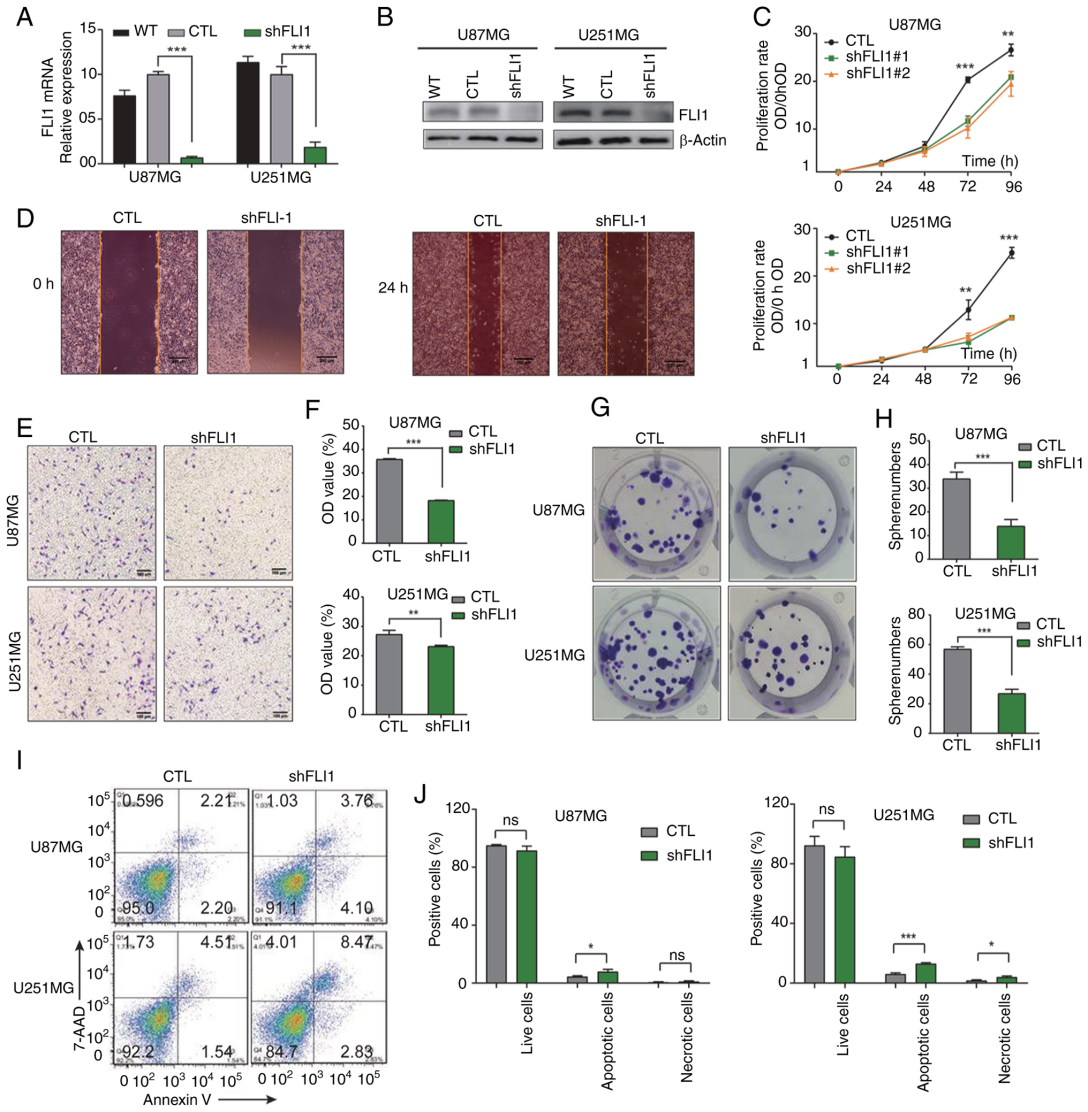
Knockdown of *FLI1* suppresses the tumor phenotypes of gliobastoma cells. (A) *FLI1* shRNA knockdown. *FLI1* was knocked down using shFLI1 in U87MG and U251MG cells. Reverse transcription quantitative PCR was performed to quantitate the *FLI1* mRNA expression level. Error bars represent the standard error of the average of three independent PCR reactions. ^***^P<0.001 as compared with the CTL. (B) *FLI1* expression by western blotting. (C) Cell proliferation as determined the Cell Counting Kit 8 assay. (D) Migration of U251MG cells. Cell migration was measured by a wound healing assay. (E) Cell invasion. Cells that invaded through the collagen-coated membrane of the Transwell after 24 h. (F) Quantitation of invaded cells. ^**^P<0.01, ^***^P<0.001 as compared with CTL. (G) Tumor sphere colonies as measured by colony-forming assay. Cell colonies were stained on day 21. (H) Quantitation of tumor sphere colonies. ^***^P<0.001 as compared with the vector control cells (CTL). (I) Cell Viability. Viability of cells after *FLI1* shRNA knockdown was measured 48 h after the treatment. (J) Quantitation of apoptotic cells and necrotic cells. ^*^P<0.05, ^***^P<0.001 as compared with CTL. shRNA, short hairpin RNA; WT, wild-type normal cells; CTL, vector control cells.

Next, the role of *FLI1* in glioblastoma cells was examined using *FLI1* shRNA and CRISPR Cas9-gRNA targeting (9). Using RT-qPCR and western blot analysis, the present study found that both shRNA (Fig. 2A and B) and Cas9 targeting (Fig. S3A) effectively decreased *FLI1* mRNA and oncoprotein production in two treated glioblastoma cell lines (U87MG and U251MG). *FLI1* depletion suppressed the malignant phenotype of U87MG and U251MG cells, including cell proliferation (Figs. 2C and S3B), migration (Fig. 2D), invasion (Figs. 2E-2F) and colony formation (Figs. 2G and H, S3C and D).

The present study further examined whether *FLI1* affected apoptosis in glioblastoma. Annexin V/7-AAD staining followed by flow cytometry was used to detect apoptosis. The proportion of apoptotic cells was significantly increased after *FLI1* shRNA knockdown in both U87MG cells and U251MG cell lines (Fig. 2I and J). Similar data were also obtained when *FLI1* was knocked down with CRISPR Cas9-gRNA (Fig. S3E and F). These results indicate that *FLI1* enhances survival and inhibits apoptosis in glioblastoma cells.

### Functional genomics identifies the FLI1 downstream pathways essential for the tumor phenotypes of glioblastoma cells

**Figure 3:**
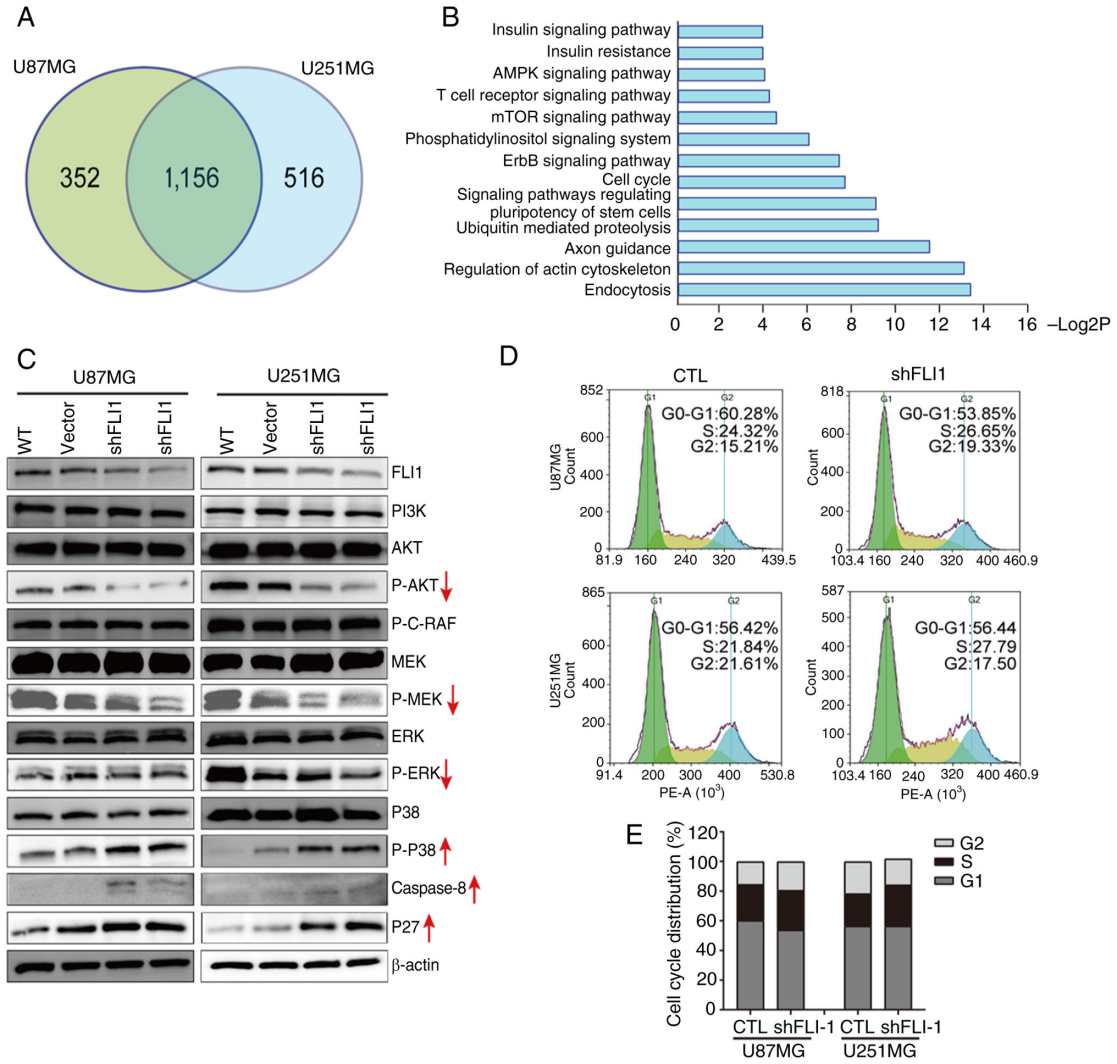
RNA-sequencing identification of *FLI1* pathways in glioma cells. (A) Venn diagram showing the overlap of shFLI1 RNA-Sequencing in U87MG and U251MG. A total of 1,156 genes were differentially expressed (>2-fold, P<0.05) after *FLI1* knockdown. (B) The KEGG pathway of *FLI1* targets (shFLI1 RNA-sequencing). (C) Western blot analysis of signal pathway targets after *FLI1* knockdown. (D) Cell cycle analysis. After *FLI1* knockdown, cell cycle was measured using FACS in *FLI1* knockdown glioblastomas cells. CTL, vector control cells. (E) Quantitation of the cell cycle. P, phosphorylation; CTL, vector control cells; KEGG, Kyoto Encyclopedia of Genes and Genomes.

Having identified the tumor-promoting role of *FLI1* in glioblastoma cells, the present study investigated the downstream molecular pathways that influence the biological behavior of glioblastoma cells. RNA-seq was performed in glioblastoma cells after *FLI1* knockdown. A total of 1,156 genes were differentially expressed in both U87MG and U251MG cells after *FLI1* knockdown (Fig. 3A). *FLI1*-related KEGG pathways identified by functional genomics analysis included 'Endocytosis', 'Regulation of actin cytoskeleton', 'Axon guidance', 'Ubiquitin mediated proteolysis', 'Signaling pathways regulating', 'Cell cycle', 'ErbB signaling pathway', 'Phosphatidylinositol signaling system', 'mTOR signaling pathway', 'T cell receptor signaling pathway' and 'AMPK signaling pathway' (Fig. 3B). Western blotting analysis revealed that *FLI1* knockdown upregulated several proteins including cyclin-related protein P27. By contrast, expression of proteins associated with cell proliferation, including P-AKT, P-MEK, P-ERK and key proteins of the AMPK were downregulated (Fig. 3C). Expression levels of Caspase 8 and p38 were upregulated, suggesting increased apoptosis. These data indicate that *FLI1* promotes glioma development by regulating multiple downstream signaling pathway targets, including proliferation-associated AKT, apoptosis-associated Caspase 8, P38 and cell cycle-related P27.

The present study used flow cytometry to analyze cell cycle in shFLI1-treated cells (Fig. 3D and E). After *FLI1* knockdown, there was an increase in the number of cells in S phase in both U251MG (27.79%) and U87MG (26.65%) cell lines as compared with the control (CTL) cells (21.84 and 24.32%). Thus, downregulation of *FLI1* results in S phase cell cycle arrest in glioblastoma cells.

### Immunoprecipitation-MS identifies ISG15 as a new molecular target of FLI1

**Figure 4:**
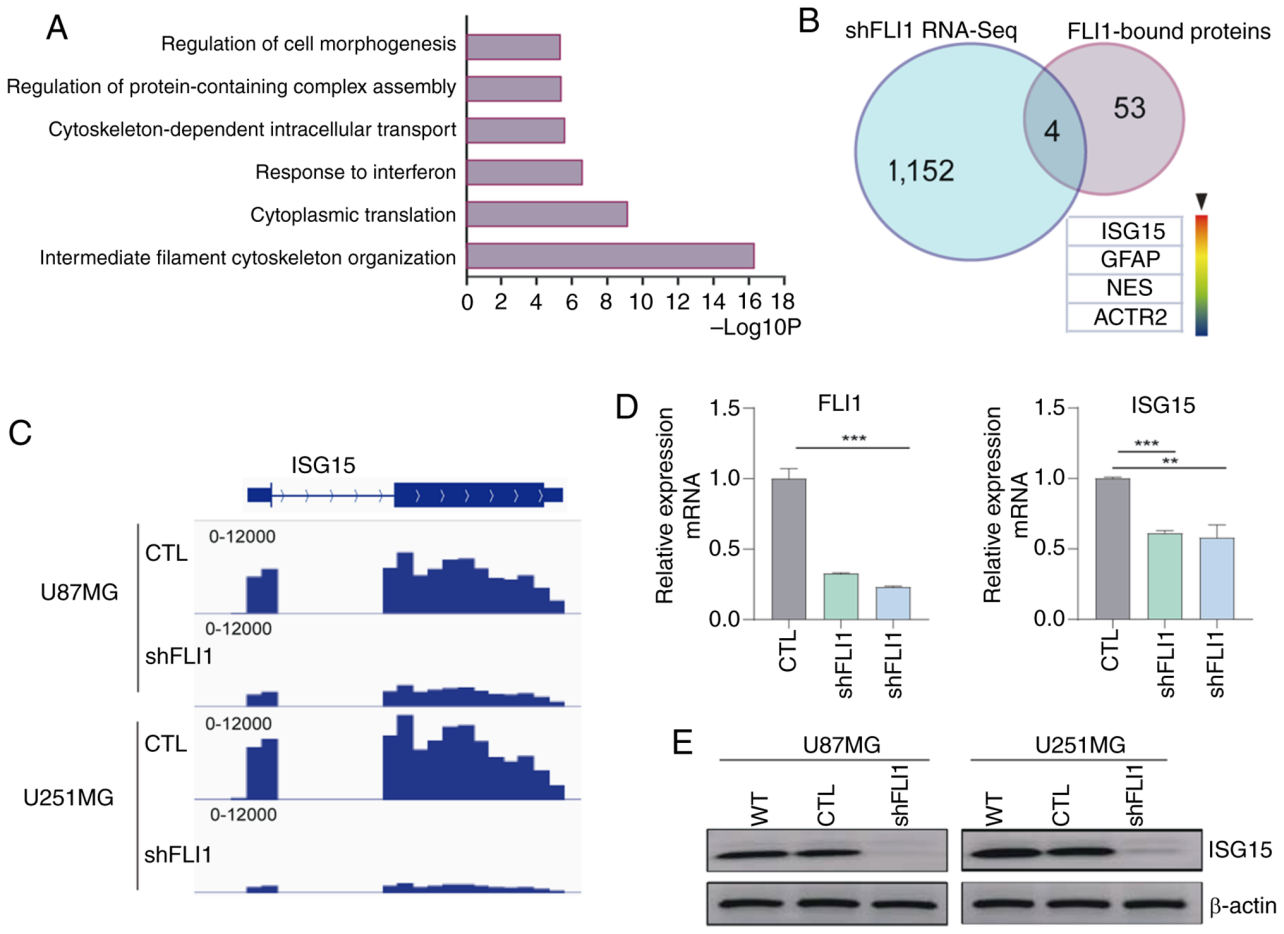
Identification of *ISG15* as a downstream target of *FLI1*. (A) Gene ontology enrichment analysis of FLI1-binding proteins. (B) Integration of the shFLI1 RNA-Sequencing and FLI1-bound protein datasets. (C) The IGV plot of *ISG15* from RNA-seq data. *ISG15* (NM_005101.4) is located at chromosome 1: (chr1:1,013,497-1,014,540). Both the shFLI1 or CTL in U87MG and U251MG cells RNA-seq Bam data were analyzed by IGV, and the Sashimi plot was acquired for ISG15. Note differential expression of ISG15 in shFLI1 and CTL cell populations. (D) *ISG15* mRNA expression in shFLI1 U87MG cells. Reverse transcription quantitative PCR was performed to quantitate the *ISG15* mRNA expression level in shFLI1 U87MG cells. The data represent the mean ± SD from three independent experiments. ^**^P<0.01, ^***^P<0.001 as compared with CTL. (E) Western blotting. The abundance of ISG15 protein was assessed by western blotting in shFLI1-treated U87MG cells. CTL, vector control cells.

The present study then utilized immunoprecipitation-MS techniques to profile target proteins that interact with FLI1 in two glioblastoma cell lines (U87MG and U251MG). GO enrichment analysis identified FLI1-binding protein targets involved in cell morphogenesis, protein-containing complex assembly, cytoskeleton-dependent intracellular transport, response to interferon, cytoplasmic translation and intermediate filament cytoskeleton organization (Fig. 4A).

To narrow down the specific *FLI1* molecule target(s), the present study integrated the shRNA-seq and FLI1-interacting protein datasets. By combining these datasets, the present study identified four candidate target molecules that are both differentially transcribed after *FLI1* knockdown and that also post-translationally interact with FLI1 protein. The interferon-stimulated gene of 15 kDa (*ISG15*) had the largest fold increment (Fig. 4B). *ISG15* was first identified as a ubiquitin-like protein (Ubl) with important immunomodulatory activity (16,27,28). RNA-seq data showed that *ISG15* is significantly reduced after *FLI1* knockdown (Fig. 4C). RT-qPCR and western blot analysis verified the downregulation of *ISG15* after *FLI1* knockdown (Fig. 4D and E), suggesting that *ISG15* could be a new molecular target of *FLI1* in glioma cells.

### ISG15 interacts with FLI1 and enhances its stability in glioblastoma cells

*ISG15* is a molecular target of *FLI1* at the transcriptional level, and its product, ISG15 protein, also interacts with FLI1 protein at the post-transcriptional level. Immunofluorescence staining shows that *FLI1* and *ISG15* proteins are co-localized (Fig. S4A). FLI1 was mainly found in the nuclear region of glioblastoma cells, while ISG15 was distributed in both the nucleus and cytoplasm; there was co-localization between FLI1 and ISG15 in the nucleus. Co-immunoprecipitation (Co-IP) confirmed the interaction between FLI1 protein and ISG15 protein in glioblastoma cells (Fig. S4B).

ISG15 has been shown to compete with ubiquitin for ubiquitin binding sites on target proteins, leading to modulation of protein degradation (29). *ISG15* overexpression stabilizes its target protein by forming an *ISG15* protein conjugate (30). The present study investigated whether the binding of ISG15 affects FLI1 protein stability in glioblastoma cells. Although *ISG15* knockdown did not affect *FLI1* at the mRNA transcript level, it significantly reduced the level of FLI1 protein (Fig. S4C and D). These data suggest that as a downstream target, *ISG15* may also affect FLI1 protein levels using a second feedback mechanism.

A cell ubiquitination assay confirmed the regulation of FLI1 protein by ubiquitination modification (Fig. S4E). Cycloheximide chase assay showed that the FLI1 protein half-life was reduced from ~9 h in the control group to ~7 h after *ISG15* knockdown (Fig. S4F). These data suggest that *ISG15* knockdown reduces the stability of the FLI1 protein while ISG15 enhances the stability of FLI1 protein through the ubiquitination pathway. Thus, the *FLI1*-*ISG15* axis is regulated through a positive feedback mechanism.

**Figure 5:**
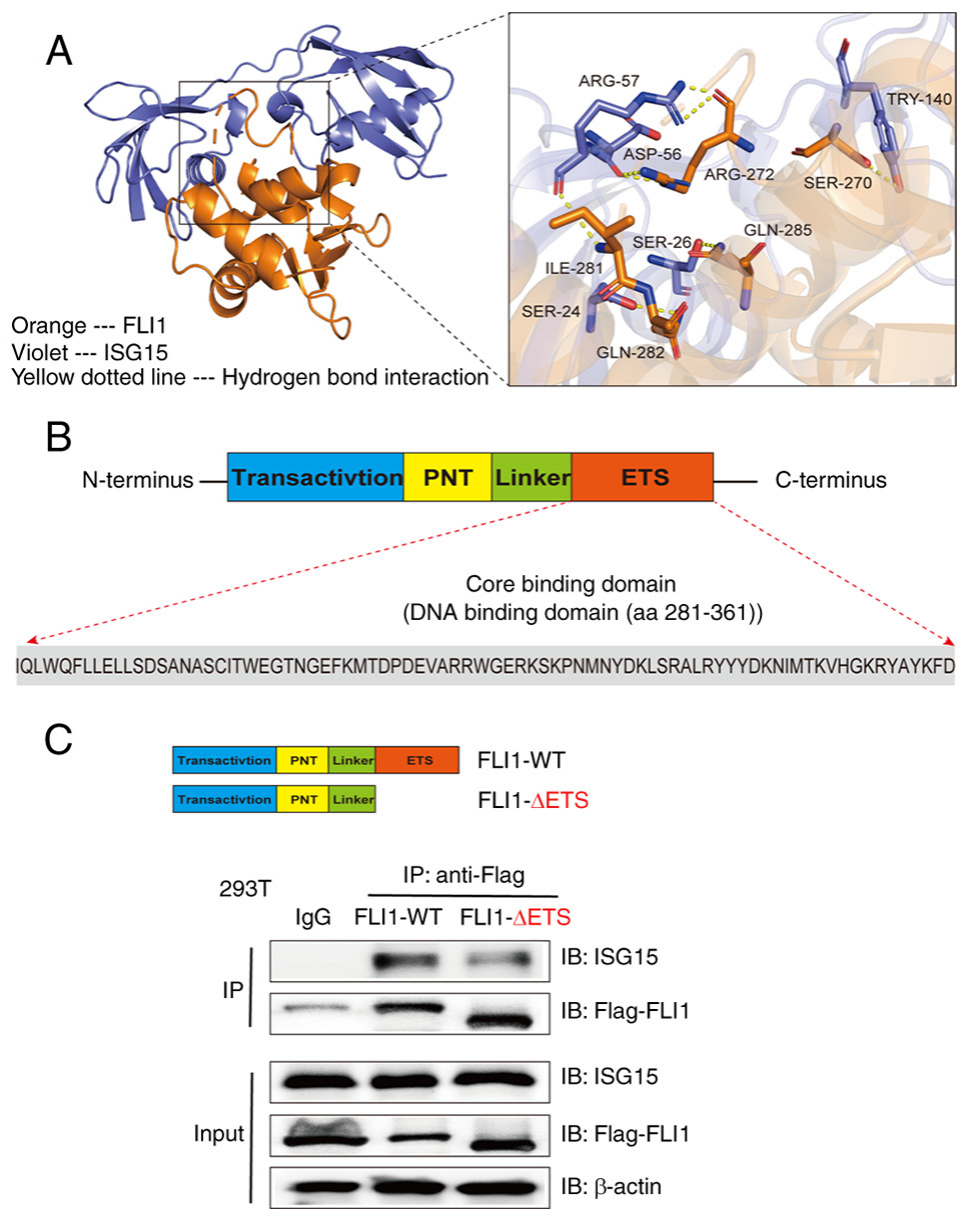
ETS of *FLI1* interaction with ISG15. (A) 3D structures showing the molecular docking analysis between FLI1 and ISG15. Orange: FLI1; Violet: ISG15; Yellow dotted line: Hydrogen bond interaction. (B) Core binding domain ETS of *FLI1*. (C) FLI1-WT-Flag and the truncation FLI1-ΔETS-Flag was expressed in 293 cells. Cells were lysed in protein extraction buffer, followed by immunoprecipitation with mouse Flag antibodies. The immunoprecipitates and cell lysates were detected with Flag antibodies and ISG15 antibodies. ETS, E26 transformation specific.

To identify the specific protein domains responsible for the interaction between FLI1 and ISG15, the present study performed molecular docking analysis using HADDOCK. The docking results were visualized and rendered as 3D structures using PyMOL. The analysis revealed a distinct binding interface between FLI1 and ISG15, with a calculated binding energy of -13.9 kcal/mol (Fig. 5A), suggesting a strong protein-protein interaction between these two proteins. Based on the predicted binding site and the structural architecture of FLI1, the present study subsequently conducted a mutation assay by deleting the FLI1 ETS domain that was predicted to interact with ISG15. Using a Co-IP assay, the present study demonstrated that the ETS domain of FLI1 is key for the interaction with ISG15 (Fig. 5B and C).

### Interaction of FLI1 with ISG15 to regulate tumor immune response in glioma cells

**Figure 6:**
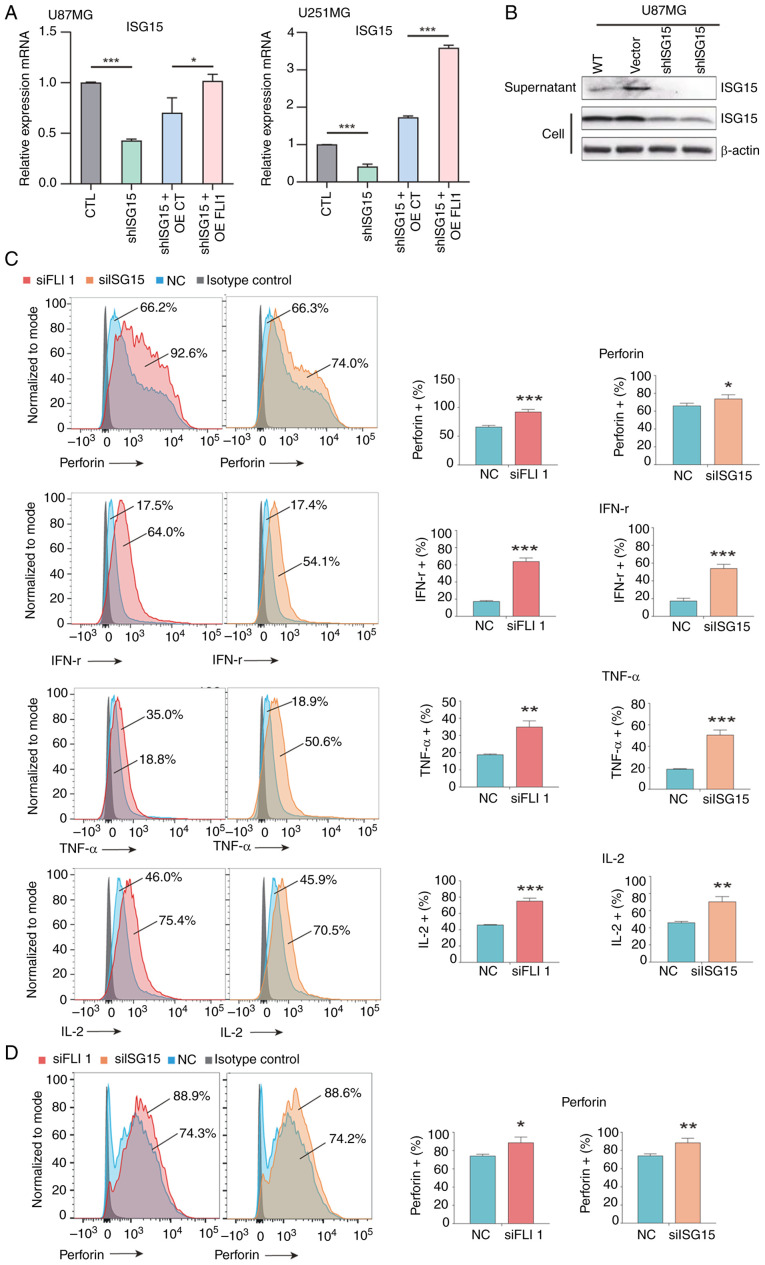
*FLI1* coordinates with *ISG15* to inhibit immune function. (A) *ISG15* rescue assay. The *ISG15* knockdown cells were incubated with cells that overexpressed *FLI1* (shISG15 + OE FLI1) or with the vector control (shISG15 + OE CT). A total of 48 h after transfection, cells were collected for the measurement of *ISG15* using quantitative PCR. The PCR value was standardized by setting the CTL control as 1. Error bars represent the standard error of the average of three independent PCR reactions. ^*^P<0.05, ^***^P<0.001 as compared with the CTL control. (B) Free extracellular *ISG15*. Western blot analysis was used to analyze the expression of free extracellular *ISG15* in the culture supernatant of glioblastoma cells. (C) Flow cytometry analysis of cytokine secretion of activated T cells induced with U251MG cells. T cells activated and amplified by CD3 antibody stimulation were incubated with siFLI1 or siISG15 U251MG cells for 4 h, followed by intracellular cytokine staining. (D) Flow cytometry analysis of perforin+ γδ T cells induced with U251MG cells. γδ T cells were incubated with siFLI1 or siISG15 U251MG cells for 4 h, followed by perforin staining. n=3 donors, ^*^P<0.05, ^**^P<0.01, ^***^P<0.001, two-tailed paired t-test. OE, overexpression; si, small interfering; NC, negative control.

*ISG15* is associated with the pathological stage, grade and prognosis of glioma (31).To further elucidate the role of the *FLI1*-*ISG15* axis, the present study investigated whether *FLI1* could affect the expression of *ISG15* in shRNA knockdown glioblastoma cells. As expected, treatment with the shRNA significantly reduced the *ISG15* expression in two glioblastoma cells. However, overexpression of *FLI1* was able to compensate for the *ISG15* downregulation induced by shRNA treatment (Fig. 6A). These data suggest that *FLI1* rescues the shRNA-induced *ISG15* knockdown.

Next, we analyzed the expression pattern of *ISG15* in clinical glioma tumor samples. *ISG15* was significantly upregulated in tumor tissues as compared with normal subjects (Fig. S5A). In addition, *ISG15* was positively associated with the histological grade of glioma (Fig. S5B). Like *FLI1*, *ISG15* expression levels were inversely associated with glioma prognosis, with the expression significantly higher in poor-prognosis glioblastomas (*IDH*-wild-type, *1p/19q* non-codel) than in favorable-prognosis oligodendrogliomas (*IDH*-mutant, *1p/19q* co-deletion) (Fig. S5C and D). The abnormally high expression of *ISG15* was associated with a malignant phenotype and poor prognosis of glioma (Fig. S5E). Immunohistochemical staining showed that *ISG15* was expressed in especially high abundance in IV glioblastoma (Fig. S5F, red arrows).

The secretable *ISG15* protein form is involved in immune regulation and plays a multifaceted role in the tumor microenvironment (16-19). Therefore, the present study measured the level of free extracellular ISG15 protein. The present study found that free ISG15 was downregulated in the *ISG15* knockdown cells (Fig. 6B). To learn whether the *FLI1*-*ISG15* axis impacts the immune environment in glioblastoma cells, the present study co-cultured *FLI1* and *ISG15* knockdown glioblastoma cells with immune cells (T cells and γδ T cells) and examined cytokine secretion in the culture media. Notably, the present study found that knockdown of *FLI1* and *ISG15* in glioblastoma cells activated T lymphocytes in this co-culture system, with significant increases in the secretion of Perforin, IFN-γ, TNF-α and IL-2 (Fig. 6C). Perforin secretion was increased in γδ T cells in the co-incubation group, but the secretion of other cytokines did not change significantly (Fig. 6D). These data suggest that *FLI1* coordinates with *ISG15* to exert immunomodulatory effects in glioblastoma cells.

### FLI1 orchestrates a unique intrachromosomal loop structure at the ISG15 locus

**Figure 7:**
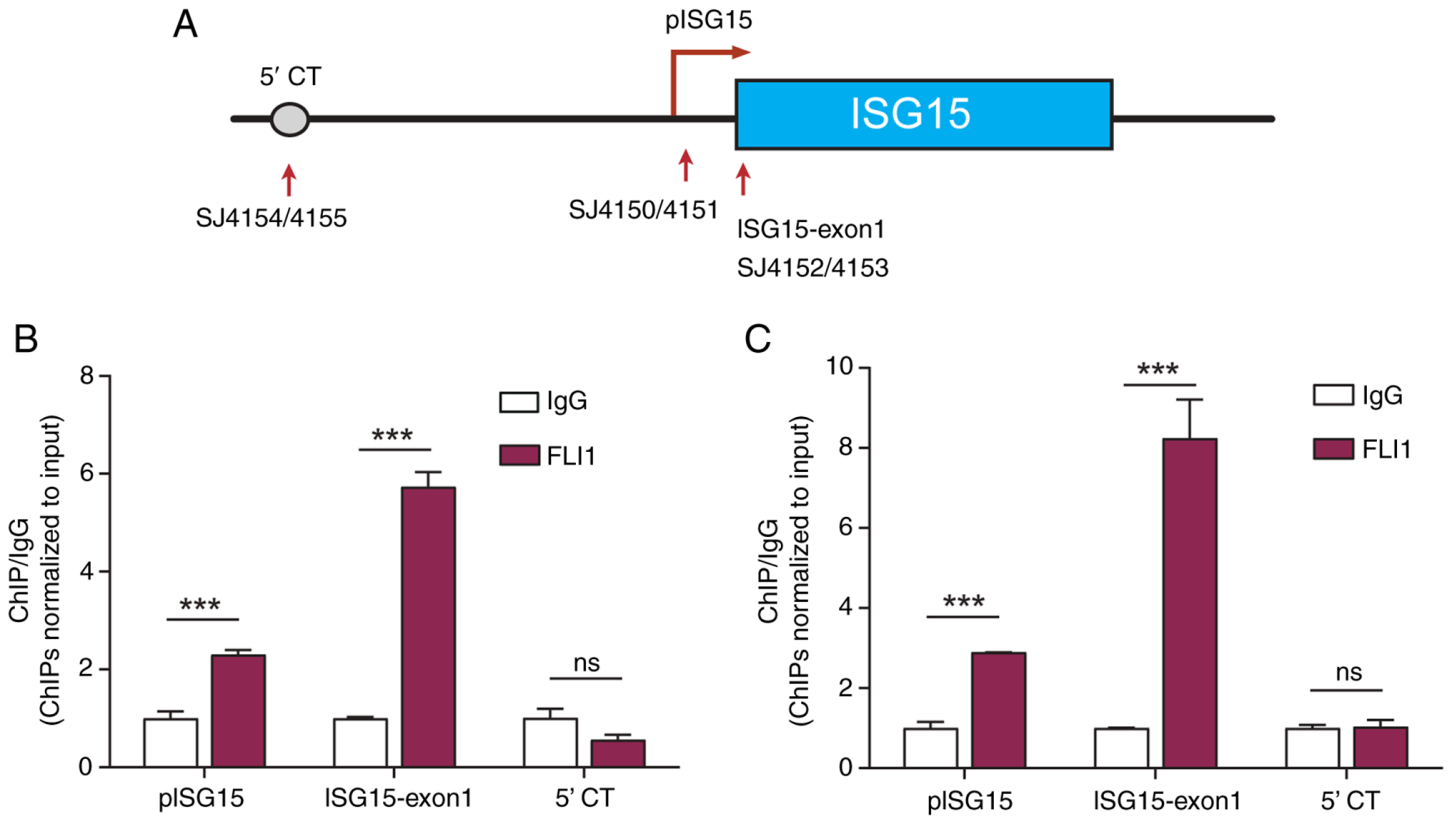
*FLI1* binding to the *ISG15* promoter. (A) Location of PCR primers used for *FLI1* binding in the *ISG15* locus. Two primer sets SJ4150/SJ4151 and SJ4152/SJ4153 were used to quantitate *FLI1* enrichment in the promoter and exon 1 of the *ISG15* gene. The primer set SJ4154/SJ4155 for the 5′-upstream site (5′ CT) was used as the negative control. The binding of *FLI1* in the *ISG15* promoter of (B) U87MG cells and (C) U251MG cells. The binding enrichment of *FLI1* in the *ISG15* promoter was measured by ChIP assay. Normal mouse IgG was used as a negative control and for normalization. The data are the mean ± SD from three independent experiments. ^***^P<0.001 as compared with CTL. CTL, control cells.

The present study then explored the molecular mechanism by which *FLI1* regulates *ISG15* in glioblastoma cells. As a member of the ETS family of transcription factors, *FLI1* is likely to function as a transcription factor to regulate *ISG15*. the present study performed a ChIP assay using a FLI1-specific antibody. Specific qPCR primers were used to detect if FLI1 binds to the *ISG15* gene, including the 5′ control region (5′ CT), the promoter (pISG15) and the exon 1 of the *ISG15* gene (*ISG15*-exon1) (Fig. 7A). Since BCL2 is a known downstream target of *FLI1* (32), the *BCL2* gene served as a positive control in the ChIP assay (Fig. S6). Analysis revealed that FLI1 bound to both the promoter and exon 1 of the *ISG15* gene (Fig. 7B and C), suggesting that *FLI1* may regulate the transcription of the *ISG15* gene through an epigenetic mechanism.

**Figure 8:**
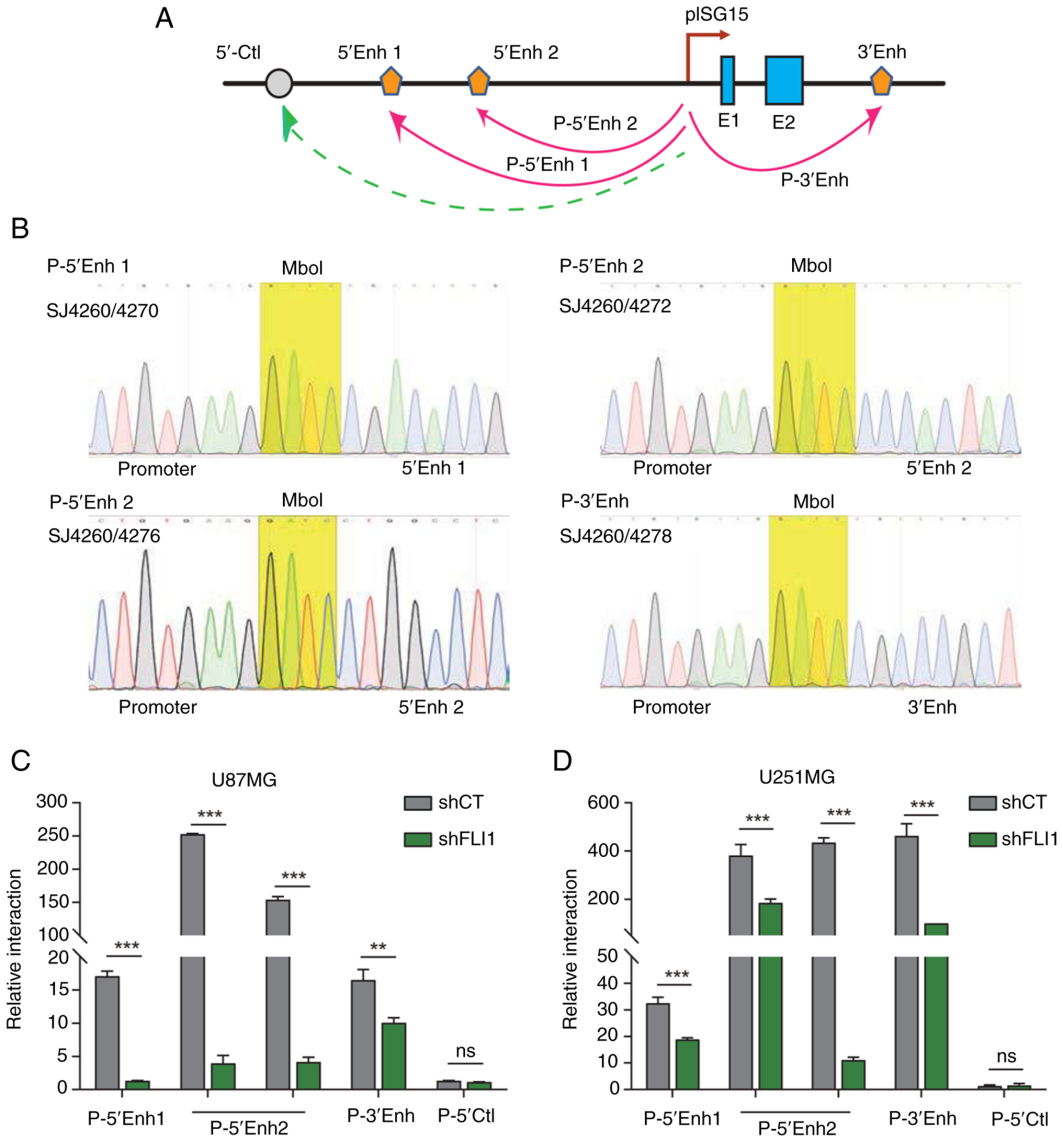
*FLI1* orchestrates intrachromosomal interactions in the *ISG15* locus. (A) Location of 3C primers used to detect the interaction between the *ISG15* promoter, 5′enhancer, and 3′enhancer. pISG15: *ISG15* promoter. E1-E2: *ISG15* exons 1-2. Enh: enhancers. Arrows: intrachromosomal interactions. (B) Sequencing of the *ISG15* intrachromosomal loop 3C products. Yellow background on the sequence: the 3C ligation product between the *MboI* restriction sites. (C) Quantitation of 3C intrachromosomal interaction signals in U87MG cells. (D) Quantitation of 3C intrachromosomal interaction signals in U251MG cells. The 3C interaction was quantitated by qPCR. shCT cells were used as the control. The data represent the mean ± SD from three independent experiments. ^**^P<0.01 and ^***^P<0.001 as compared with the control group (shCT). ns, no significance; sh, short hairpin; Ctl, control.

The three-dimensional structure among gene regulatory elements precisely regulates gene expression (26,33). The present study used a 3C approach (11,26,34) to compare intrachromosomal loop structures at the *ISG15* locus and determined if *FLI1* regulates *ISG15* by altering local chromatin structure in glioblastoma cells. 3C primers were designed at various regions of *ISG15*, including the 5′-enhancer, the promoter, the 3′-enhancer, and the 5′ control region (Fig. 8A). The present study detected the presence of the intrachromosomal loops between the promoter and enhancers and verified these high order structures by DNA sequencing of the 3C PCR products, showing the *MboI* junction sites flanked by *ISG15* promoter and enhancer sequences (Fig. 8B). Of note, *FLI1* knockdown significantly reduced these chromatin structures, as compared with the shCT control (Fig. 8C and D). These data indicate that there is an intrachromosomal loop orchestrated by FLI1 at the *ISG15* locus that juxtaposes the upstream and downstream enhancers to the promoter of the *ISG15* gene, thereby activating *ISG15* gene in *cis*. *FLI1* is involved in the formation and maintenance of this *ISG15* intrachromosomal loop structure.

### FLI1 maintains an active chromatin structure at the ISG15 promoter characterized by DNA hypomethylation and histone acetylation

**Figure 9:**
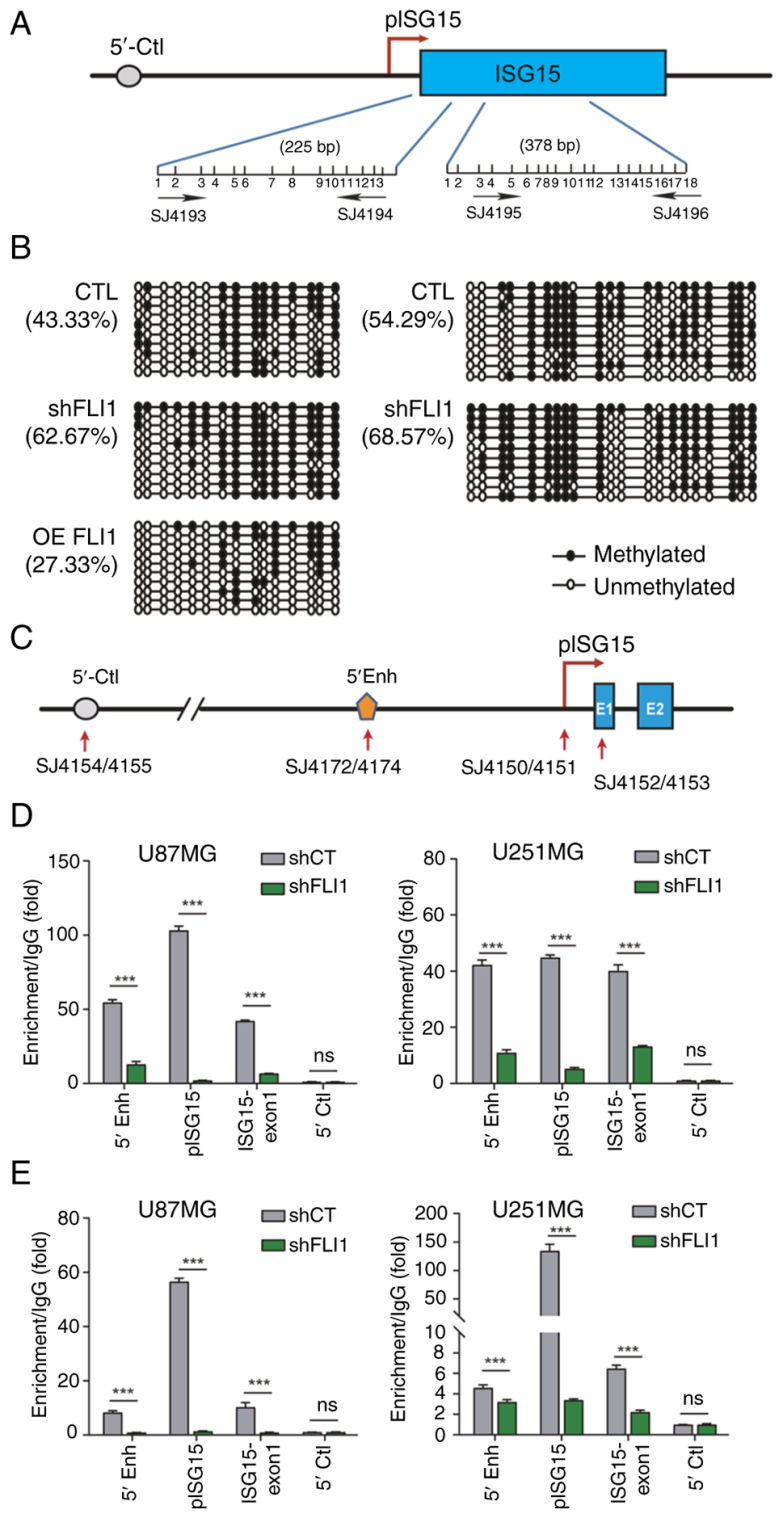
*FLI1* regulates *ISG15* through epigenetic pathways. (A) Schematic diagram of CpG sites in the *ISG15*. Locations of PCR primers are indicated by numbered arrows. Vertical lines: Location of CpGs. pISG15: *ISG15* promoter. Two CpG islands in the promoter and the first exon of *ISG15* were selected for the measurement of DNA methylation. (B) Alteration of DNA methylation at the *ISG15* site in U251MG cells. U251MG cells were transfected with CTL, shFLI1 and *FLI1*. Cells were collected for measurement of DNA methylation by sodium bisulfite sequencing. Solid dot: Methylated CpG islands; Open dot: unmethylated CpG islands. Numbers in the parenthesis: the percentage of total methylated CpGs over the CpGs in the sequencing. Each line represents the sequence for one clone. A total of 10 clones were sequenced for each group. (C) Location of PCR primers used for H3K9AC and H3K27AC in the *ISG15* locus. Primer sets SJ4150/SJ4151, SJ4152/SJ4153 and SJ4172/SJ4174 were used to quantitate the *FLI1* binding enrichment in the promoter, exon 1 and enhancer of the *ISG15* gene, respectively. The primer set SJ4154/SJ4155 for the 5′-upstream region (5′Ctl) was used as the negative control. Quantitation of histone modification (D) H3K9AC and (E) H3K27AC in the *ISG15* promoter of U87MG cells (left) and U251MG cells (right). H3K9AC and H3K27AC modifications in the *ISG15* promoter were measured by ChIP assay. Normal rabbit IgG was used as a negative control and for normalization. The data are the mean ± SD from three independent experiments. ^***^P<0.001 as compared with CTL. CTL, control vector; sh, short hairpin.

The present study next examined whether the FLI1-mediated chromatin 3D structure impacts the transcriptional activity of *ISG15* altering DNA methylation and histone modifications. First, we collected DNA from U251MG cells after *FLI1* overexpression or knockdown, and used sodium bisulfite sequencing to examine the methylation status of CpG islands in the *ISG15* gene (Fig. 9A). When *FLI1* was knocked down, the methylation ratio of the *ISG15* promoter increased from 43.33 to 62.67% in the *ISG15* promoter (Fig. 9B). By contrast, *FLI1* overexpression induced hypomethylation (27.33%) in the *ISG15* promoter (Fig. 9B, left bottom panel). Similarly, *FLI1* also induced *ISG15* DNA demethylation in U87MG cells (Fig. S7).

To investigate whether *FLI1* regulates *ISG15* expression through altered histone acetylation, the present study used chromatin immunoprecipitation qPCR (ChIP-qPCR) to quantify changes in H3K9AC and H3K27AC histone marks in the *ISG15* promoter of cells before and after knockdown of *FLI1*. H3K9AC and H3K27AC histone marks were significantly reduced by >10-fold in *FLI1* knockdown cells (Fig. 9C-E).

The data of the present study suggests that FLI1 binds to *ISG15* regulatory regions, where it regulates *ISG15* gene activity by orchestrating an active chromatin looping spatial structure, with DNA hypomethylation and enhanced histone H3K9 and H3K27acetylation. *FLI1* activates the *ISG15* gene and promotes *ISG15* transcription in glioblastoma cells through these epigenetic mechanisms.

## Discussion

**Figure 10:**
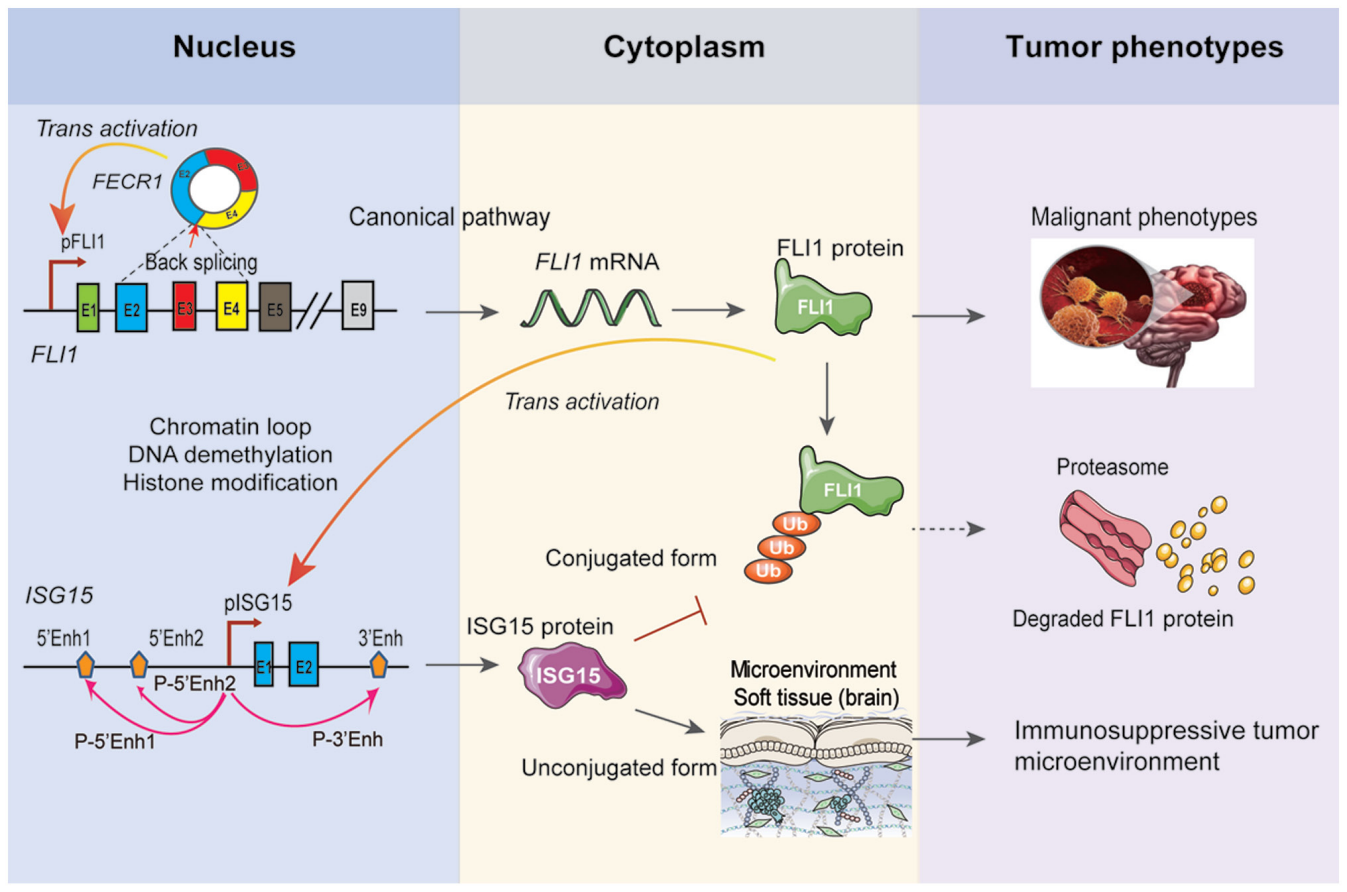
The exonic circRNA *FECR1-FLI1-ISG15* crosstalk circuitry. *FLI1* not only drives tumor growth through the canonical oncoprotein pathway, but it also influences glioblastoma development at multiple levels. In addition to its linear mRNA, the *FLI1* gene also transcribes an exonic circRNA *FECR1*, composed of FLI1 exons 4-2-3 that is produced by back splicing. Through a positive feedback mechanism, exonic circRNA *FECR1* can upregulate its parental gene transcription. *FLI1* protein acts as a key transcription factor after entering the nucleus where it promotes the transcriptional expression of *ISG15* through epigenetic mechanisms. A positive feedback regulatory loop between *FLI1* and *ISG15* contributes to the malignant and immunosuppressive tumor microenvironment in glioblastoma.

Glioma, the most prevalent primary cancer of the brain, has a high degree of malignancy and a poor prognosis, and there are no targeted therapeutics in clinical practice. The present study shown that *FLI1*, an oncogenic driver of hematological malignancies, may serve as a potential therapeutic target for gliomas (Fig. 10). *FLI1* is upregulated in glioma through a positive feedback loop from its exonic circRNA *FECR1* at the transcriptional level. The overexpression of *FLI1* promotes tumor growth and is associated with the malignant phenotype and poor prognosis of glioblastoma. FLI1 activates the *ISG15* gene, a paracrine factor that reprograms and maintains an immunosuppressive tumor microenvironment. The present study has shown that FLI1 orchestrates an active chromatin conformational structure between the *ISG15* gene promotor and its enhancer, characterized by DNA hypomethylation and histone H3K9 and H3K27 acetylation. This oncogenic diathesis is self-magnifying, as the ISG15 protein also interacts with the FLI1 protein and thereby enhances FLI1 stability by blocking its degradation through the ubiquitination pathway. Thus, *FLI1* is regulated by two crosstalk loops in glioblastoma cells. The exonic circRNA *FECR1* forms the first feedback loop as it upregulates the *FLI1* gene at the transcriptional level. In addition, the downstream target ISG15 protein mediates a second feedback loop, stabilizing FLI1 protein at the posttranscriptional level. Through these two feedback regulatory loops, *FLI1* is overexpressed in glioblastoma, and it coordinates with *ISG15* to contribute to glioblastoma malignancy and immunosuppression of the tumor microenvironment. As a result, targeting these *FLI1-ISG15* crosstalk loops may provide a multifaceted basis for treating glioblastoma (Fig. 10).

*FLI1* is highly expressed in a variety of solid tumors (6,8-11). *FLI1* is considered to be an oncogene, regulating pathways associated with proliferation, angiogenesis, genomic instability, inhibition of apoptosis and differentiation (35,36). The present study has demonstrated that *FLI1* is highly expressed in glioblastoma and that its expression is positively associated with the malignant grade and poor prognosis. the present study showed that *FLI1* exonic circRNA *FECR1* is an RNA component of the *FLI1* promoter complex, and that exonic circRNA *FECR1* activates gene activity through epigenetic mechanisms. Exonic circRNA *FECR1* is expressed in glioma. Previously, we demonstrated that exonic circRNA *FECR1* physically interacted with its host gene *FLI1* promoter, where it serves as a scaffolding factor to recruit the DNA demethylase TET1. By tethering TET1 to the *FLI1* promoter, exonic circRNA *FECR1* promoted active DNA demethylation at the promoter and upregulated the host *FLI1* (11). The present study demonstrates that as a feedback regulator, exonic circRNA *FECR1* has a positive regulatory function on the parent *FLI1* gene activity. Ectopic overexpression of exonic circRNA *FECR1* significantly activates *FLI1* and enhances its expression. By contrast, exonic circRNA *FECR1* knockdown significantly reduces the level of *FLI1* in glioblastoma cells. Targeting exonic circRNA *FECR1* could interrupt this positive feedback regulatory loop and inhibit *FLI1* at the transcriptional level.

The glioblastoma microenvironment is a highly suppressed milieu of immune cells leading to a high susceptibility to relapse and metastasis (1,2,37-39). *FLI1* can affect the function of immune cells by regulating cytokines and chemokines that modulate immune cell development, activation, migration and depletion (13-15). Previous studies have shown that *FLI1* is associated with the etiology of several autoimmune diseases, including systemic sclerosis and systemic lupus erythematosus (40-42). A previous study identified T cell *FLI1* as a factor modulating CD8^+^ T cell effector differentiation with limited effects on T cell exhaustion (43). CD8^+^T cells lacking *FLI1* provide improved protection against a variety of infections and tumors (43). The present study demonstrates that *FLI1* knockdown in glioblastoma cells increases cytokine secretion from co-cultured T cells, underscoring the key role of tumor-intrinsic *FLI1* in inhibiting T cytokine secretion. Thus, *FLI1* is a key factor that impairs T cell anti-tumor immunity in the immunosuppressive tumor microenvironment (44).

*FLI1* directly regulates gene expression as a transcription factor, and it also indirectly affects the expression pattern of genes through epigenetic mechanisms (45). *FLI1* can alter chromatin structure by interacting with chromatin remodeling complexes, thereby affecting gene accessibility and transcriptional activity (45). In Ewing sarcoma, the EWS-FLI1 fusion protein changes the state of local chromatin by binding to specific DNA sequences, allowing the expression of previously silenced genes, or preventing the normal expression of certain genes and these changes contribute to tumor growth and spread (46). The study identified *ISG15* as a new target of *FLI1*. The regulation of *ISG15* by *FLI1* has not been previously reported. The present data suggest that aberrant *FLI1* overexpression in glioblastoma cells epigenetically activates the transcriptional expression of *ISG15*. *FLI1* coordinates the formation and maintenance of an intrachromosomal 3D structure in the *ISG15* locus. *FLI1* binds to the *ISG15* promoter and juxtaposes it to the upstream and downstream enhancers, forming an active chromatin complex around the *ISG15* promoter that is characterized by DNA hypomethylation and increased histone H3K9 and H3K27 acetylation. These findings suggest that *FLI1* is a potential upstream regulator of *ISG15*. Working together, these two genes contribute to the immunosuppressive tumor microenvironment.

ISG15, the product of IFN-stimulated gene 15, was first identified as a ubiquitin-like protein (Ubl). It functions both as an unconjugated form and also as a form covalently conjugated onto a target protein. *ISG15* and the members of the enzymatic cascade mediate ISG15 conjugation (ISGylation) as induced by type I interferons involved in host antiviral immunity, anti-bacterial immunity, and autoimmunity (47). *ISG15* and ISGylation are also implicated in a variety of pivotal cellular processes, involving protein translation (48), autophagy (49,50), exosome secretion (51), DNA repair (52) and immune modulation (16,27,28). The expression of *ISG15* and enzymes that catalyze ISGylation and deISGylation are dysregulated in several types of cancer, including bladder, breast and prostate cancer (31,53,54). *ISG15* is also involved in immune regulation and plays an important role in several aspects in the tumor microenvironment and in tumor cells (16-19). Treatment of cultured cells with exogenous soluble ISG15 induces PBMC to produce anti-inflammatory IL-10, which exerts immunosuppressive properties in the tumor microenvironment (55). *ISG15* also activates innate and adaptive immune responses (52,56) and ISGylation processes (47,57,58) of multiple proteins that mediate central immune signaling pathways such as NF-kB, JNK and IRF-3 (59-61). Extracellular unconjugated ISG15 functions as a cytokine to modulate immune responses (19,62-66). Free ISG15 plays different roles in various cellular processes ranging from cancer and defense against viral infections to activation vs. inhibition of immune cells (16,18,52). Free ISG15 also upregulates programmed cell death ligand 1 (PD-L1) in PDAC cells and reduces tumor-infiltrating CD8+ T cells, thereby promoting immunosuppressive TME. These actions of ISG15 occur independently of IFN-γ (67). For example, blockade of LFA-1 or SRC signaling by small molecule inhibitors or neutralization with anti-CCL18 antibodies, inhibited LFA-1-SFK-CCL18 pathway activation in ISG15-treated macrophages (68,69). ISG15 has been found to inhibit the immune function of CD8+ T cells as tumor cells can actively or passively affect the microenvironment by secreting ISG15, thereby inhibiting the immunotoxicity of cytotoxic T lymphocytes and natural killer cells (67,69). In addition, ISG15 can also participate in SRC kinase family (SFK) signaling through its interaction with its receptor LFA-1, resulting in the secretion of CCL18 and by this mechanism, ISG15 can induce M2 polarization in macrophages thereby affecting the tumor microenvironment (68,69). The present study demonstrates that ISG15 interacts with FLI1 protein and enhances FLI1 protein stability through the ubiquitination pathway. Thus, as a downstream target, ISG15 also forms a second positive feedback loop to contribute to *FLI1* overexpression in glioblastoma cells. In addition, The present study detected the functional free form of ISG15 in the cell culture supernatant of glioblastoma cells. Treatment of cells with *ISG15* shRNA reduced the level of free ISG15 in cell supernatants. However, overexpression of *FLI1* was able to reverse this shRNA-induced effect. Thus, *FLI1* and *ISG15* coordinate to establish a positive feedback circuitry in glioblastoma cells. Targeting either *FLI1* or *ISG15* in this positive feedback loop affects cytokine secretion in co-cultured immune cells, suggesting an intervention strategy for altering the tumor microenvironment and enhancing the cytotoxicity of the immune system to tumor cells.

However, the present study acknowledges that the lack of *in vivo* validation using mouse models is a limitation. Although the analysis of human clinical samples and independent public datasets suggests a key role of the *FLI1-ISG15* axis, its clinical relevance using animal models remain essential for fully elucidating the complex tumor microenvironment and systemic effects. Therefore, it is necessary to target the *FLI1-ISG15* axis in orthotopic glioma mouse models or patient-derived xenografts. These *in vivo* studies will help to validate the role of the *FLI1-ISG15* axis on tumor growth and immune suppression and to explore potential therapeutic interventions. In addition, due to the small sample size with an uneven distribution of patients across different treatment subgroups in the hospital cohort samples, the present study was not able to perform survival analyses stratified by chemotherapy, radiotherapy or combined modalities. Future studies with larger cohort samples are needed for these survival analyses.

In conclusion, the present study delineates the molecular mechanisms underlying the role of *FLI1* in glioblastoma at multiple levels. *FLI1* not only drives tumor development through the conventional oncoprotein pathway, but it also regulates the malignant phenotype and immunosuppression in glioblastoma by epigenetic pathways. There is a unique regulatory crosstalk interaction that contributes to the overexpression of *FLI1* in glioblastoma. First, *FLI1* is auto-regulated by its exonic circRNA *FECR1* through a positive feedback loop. FLI1 protein then activates the gene target *ISG15*, a tumor microenvironment remodeling factor. Finally, ISG15 also interacts with FLI1 protein and enhances its stability by blocking the ubiquitination-induced degradation. Through this crosstalk, *FLI1* and *ISG15* synergistically participate in the regulation of the tumor immune microenvironment. Targeting *FLI1-ISG15* regulatory loops may provide a promising avenue for developing new therapeutics for patients with glioblastoma.

## Supplementary Data



## Data Availability

The data generated in the present study may be requested from the corresponding author. RNA-seq data generated in the present study have been deposited in NIH GEO databases under accession number of GSE316716 (https://www.ncbi.nlm.nih.gov/geo/query/acc.cgi?acc=GSE316716). The data of LC-MS/MS have been deposited in the OMIX, China National Center for Bioinformation / Beijing Institute of Genomics, Chinese Academy of Sciences with the accession no. OMIX016920 (https://ngdc.cncb.ac.cn/omix/release/OMIX016920) (70,71).
