# Supplementary material for: FLI1 enhances the malignant phenotype of glioma cells and exerts immunomodulatory effects through feedback crosstalk with exonic circRNA FECR1 and interferon-induced ISG15
Source: Int J Mol Med. 2026 Jul 1;58(3):241. doi: 10.3892/ijmm.2026.5912 (PMC13354330; doi:10.3892/ijmm.2026.5912)

Figure S1. The exonic circRNA *FECR1-FLI1* positive feedback loop in glioma cells. (A) Upregulation of *FLI1* by its exonic circRNA *FECR1* through a positive feedback mechanism. Exonic circRNA *FECR1* is formed by back splicing of *FLI1* exon 4 to exon 2. After binding to the *FLI1* promoter, exonic circRNA *FECR1* recruits TET1 demethylase and induces DNA demethylation in the promoter CpG islands. Additionally, exonic circRNA *FECR1* also inhibits DNA methyltransferase DNMT1 to induce DNA methylation. Using this positive feedback mechanism, exonic circRNA *FECR1* regulates *FLI1* transcription in tumors [11]. (B) Exonic circRNA *FECR1* expression in U87MG cells. Exonic circRNA *FECR1* is formed by back splicing of FLI1 exons E4-E2-E3. The back splicing site of exonic circRNA *FECR1* was verified by reverse transcription-quantitative PCR sequencing. (C) RNA fluorescence in situ hybridization (FISH) for exonic circRNA *FECR1*. Nuclei were stained with DAPI. 18S as control. Scale bar, 10 $\mu$m. BF, bright-field.

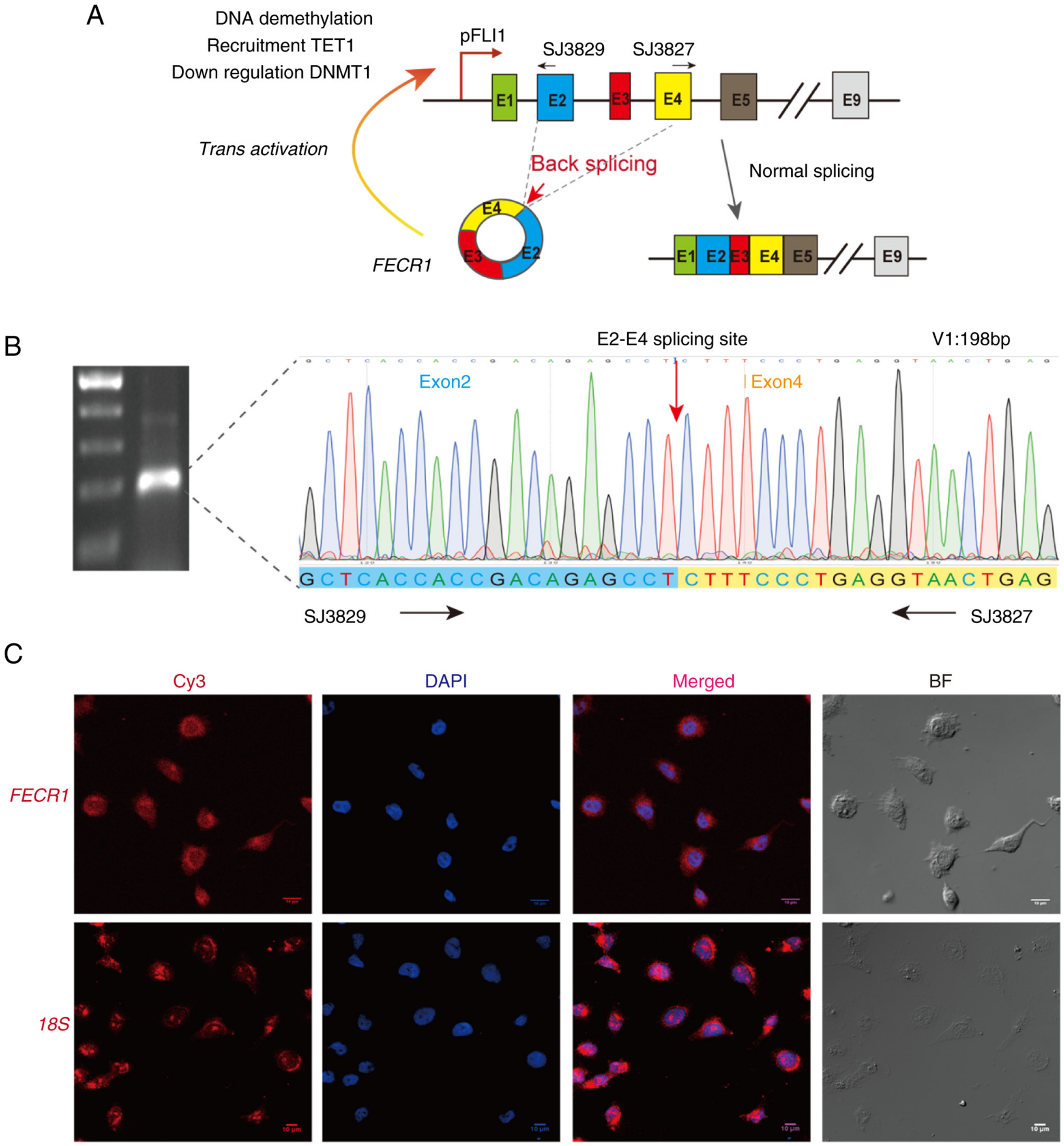

Figure S2. Exonic circRNA *FECR1* upregulates *FLI1* in glioma cells. (A) Location of PCR primers used for exonic circRNA *FECR1* binding *FLI1* (top). Binding of exonic circRNA *FECR1* in the *FLI1* locus (bottom). Two primer sets P1, P2 and E1 was used to quantitate *FLI1* in promoter and exon 1 of *FLI1*. The primer set 5'Ctl for the promoter upstream site was used as the negative control. Exonic circRNA *FECR1* was (B) overexpressed and (C) knocked down in U87MG cells. Reverse transcription PCR was performed to quantitate the exonic circRNA *FECR1* and *FLI1* expression level (Left). Error bars represent the standard error of the average of three independent PCR reactions. *P<0.05, ***P<0.001 as compared with CTL. The abundance of FLI1 protein was assessed by western blotting (Right). CTL, control; OE, overexpression; sh, short hairpin.

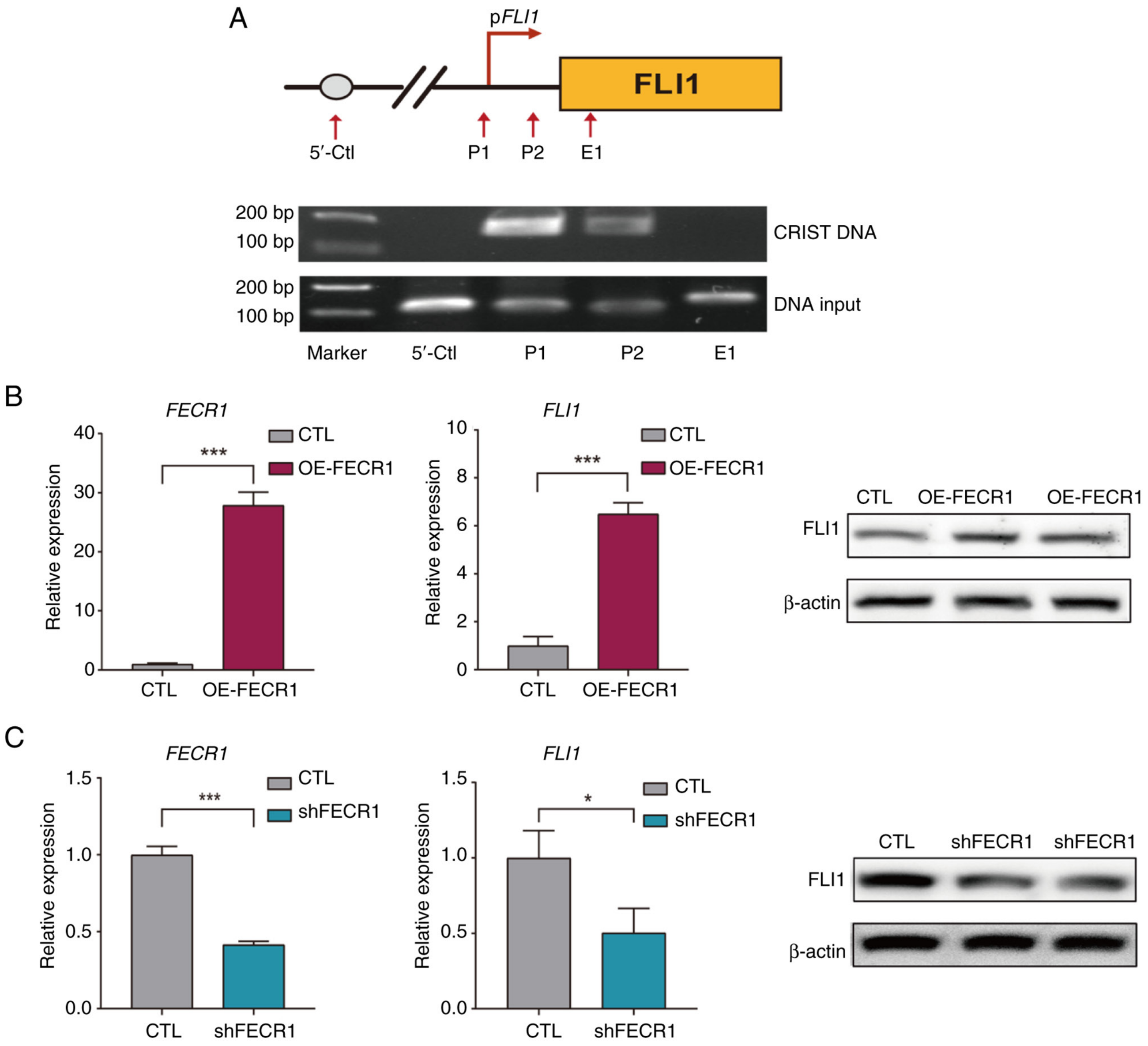

Figure S3. Cas9 *FLI1* knockdown affects tumor phenotypes in glioma cells. (A) FLI1 protein was assessed by western blotting in Cas9 FLI1 knockdown glioblastoma cells. FLI1 was knocked down using Cas9 FLI1 gRNA in U87MG and U251MG cells. (B) Cas9 *FLI1* cell proliferation was determined using the Cell Counting Kit 8 assay. (C) Cas9 *FLI1* tumor sphere colonies as measured by colony-forming assay. Cell colonies were stained on day 21. (D) Quantitation of tumor sphere colonies. $^{***}$P<0.001 as compared with the CTL. (E) Viability of cells after *FLI1* knockdown by Cas9 *FLI1* gRNA in U87MG and U251MG cells. (F) Quantitation of the apoptotic cells and necrotic cells. $^{**}$P<0.01, $^{***}$P<0.001 as compared with the CTLs. CTL, vector control cells.

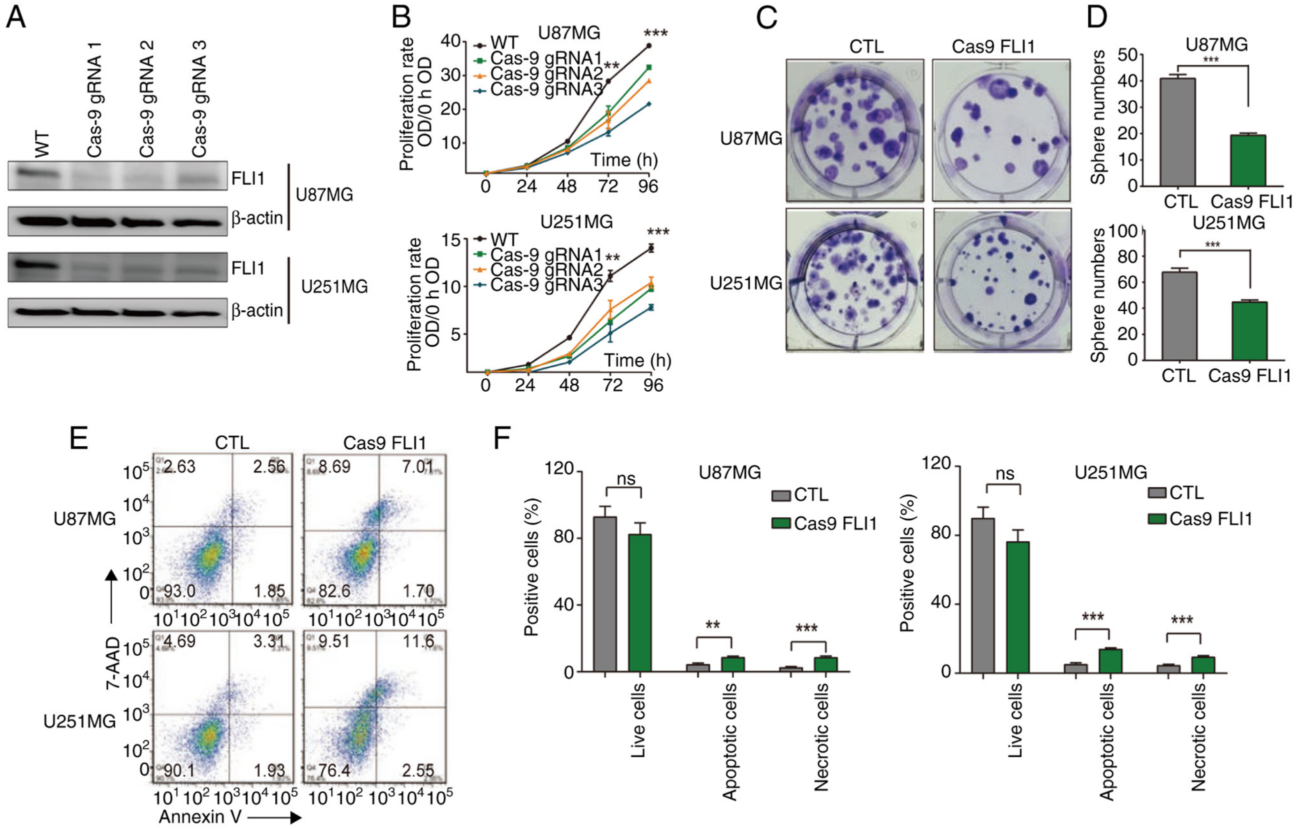

Figure S4. *ISG15* stabilizes *FLI1* in glioma cells using a positive feedback mechanism. (A) Fluorescent microscope image showing the subcellular localization of GFP-FLI1 and *ISG15*; DAPI was used to stain the nucleus. (B) Representative western blots for *FLI1* and *ISG15* immunoprecipitated by FLI1-specific, ISG15-specific and immunoglobulin G (IgG: negative control) antibodies. (C) *ISG15* was knocked down using shISG15 in U87MG. Reverse transcription quantitative PCR was performed to quantitate the *ISG15* and *FLI1* mRNA expression level. Error bars represent the standard error of the average of three independent PCR reactions. $^{***}P<0.001$ as compared with CTL. ns: Not statistically significant. (D) The abundance of FLI1 and ISG15 protein was assessed by western blotting. Note, *FLI1* transcript levels were not affected after *ISG15* knockdown, while translation levels were significantly lower. (E) FLI1-Flag was co-expressed with HA-ubiquitin in 293 cells. Cell lysates were lysed in denaturing protein extraction buffer, followed by immunoprecipitation with mouse Flag antibodies. The immunoprecipitates and cell lysates were detected with rabbit Flag antibodies and HA antibodies. (F) A cycloheximide (CHX) chase assay for FLI1 expression in U87MG cells. CTL, vector control cells; sh, short hairpin; WCL, whole cell lysate. BF, bright-field.

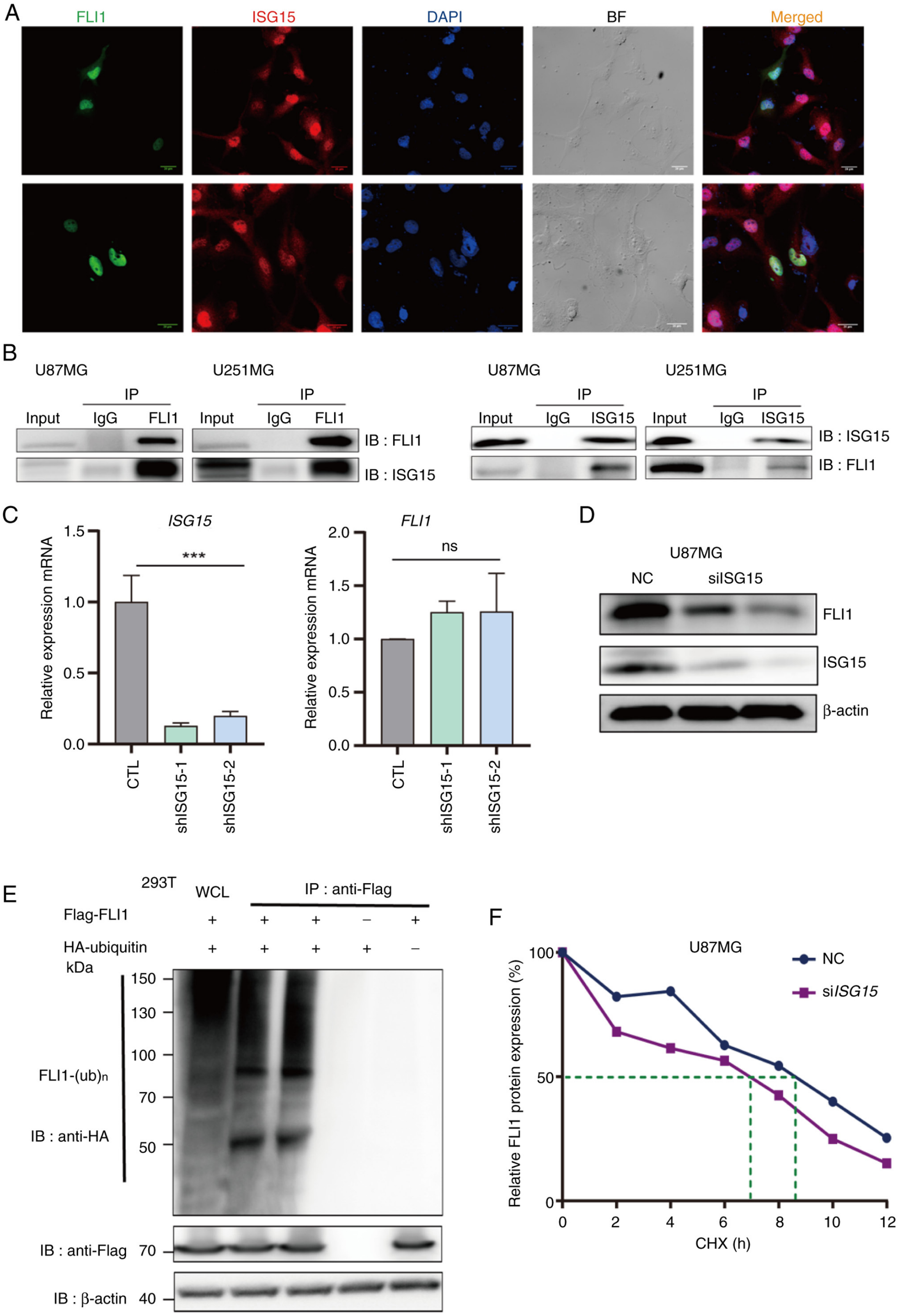

Figure S5. *ISG15* is associated with the development of glioma. (A) *ISG15* was highly expressed in glioma compared with normal brain specimens (P<0.05). (B) Histological grade of glioma. The expression level of *ISG15* was positively correlated with the histological grade of gliomas. One-way ANOVA demonstrated that *ISG15* levels varied significantly among the different histological grades ($P=1.3x10^{-17}$). (C) *ISG15* expression in *IDH* mutation and *IDH* wild-type cells. One-way ANOVA showed a highly significant difference in *ISG15* levels between the two groups ($P=6.7x10^{-13}$). (D) *ISG15* expression in *1p/19q* co-deletion and Non-codel cells. *ISG15* levels were significantly lower in the *1p/19q* co-deletion group compared with the Non-codel group ($P=3.1x10^{-15}$). (E) Overall survival. Elevated *ISG15* expression was associated with reduced OS (P=0.0001). (F) Overexpression of *ISG15* in glioma tissues. *ISG15* expression was quantitated by immunohistochemical staining and was evaluated as the expression score. Red arrow: Dark brown immunohistochemical staining of *ISG15* oncoprotein. $^{*}$P<0.05 in *IV* glioma tissues as compared with *II/III* glioma tissues. Scale bar, 200 $\mu$m. The gene expression data in Fig. S5A were generated from the GEPIA web server http://gepia.cancer-pku.cn/index.html. Histological grade, molecular features and survival data in Figures S5B-S5E were retrieved from Chinese Glioma Genome Atlas https://www.cgga.org.cn/index.jsp. NTC, non-tumoral controls; T, tumors; N, number, *IDH,* isocitrate dehydrogenase.

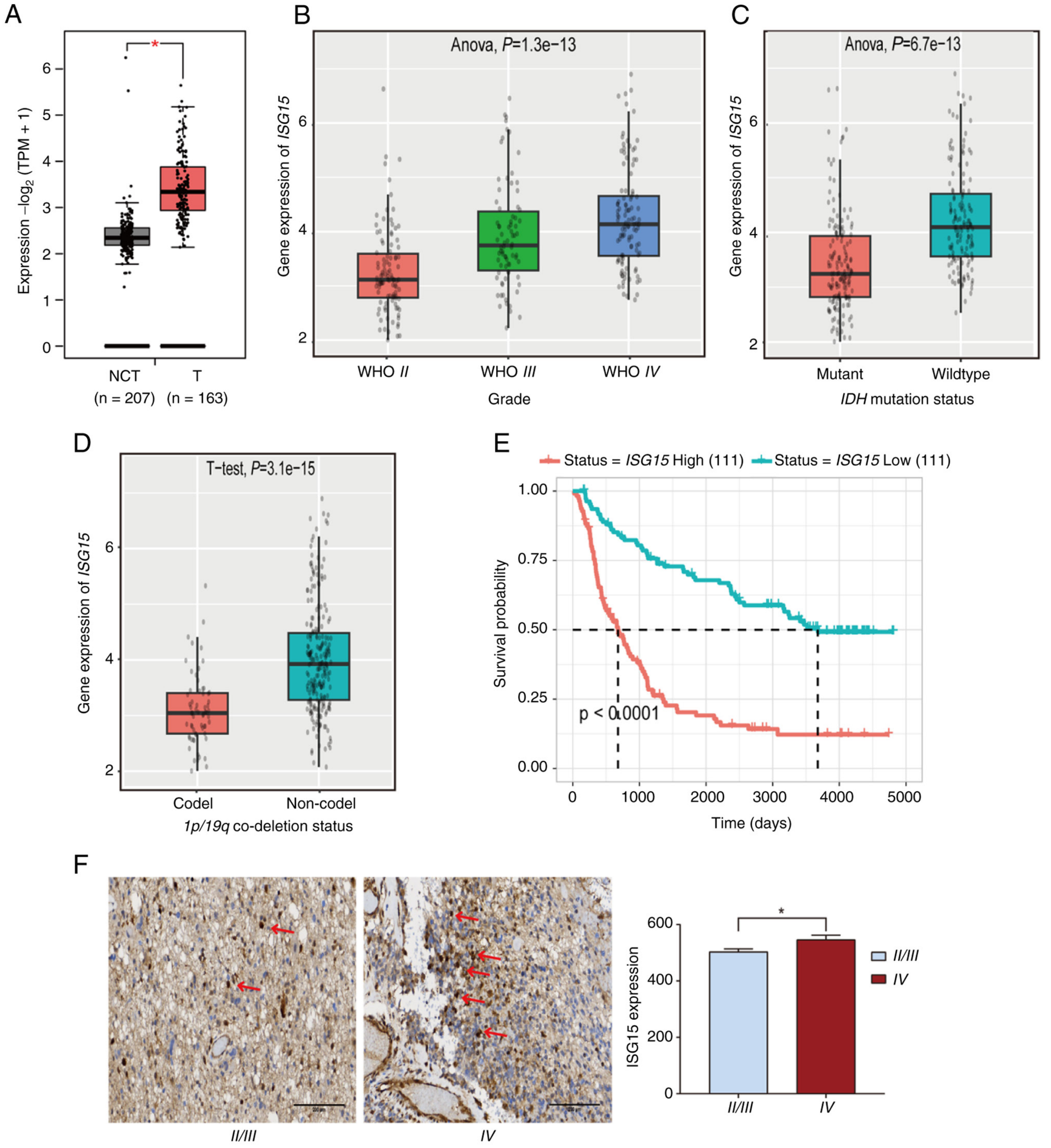

Figure S6. *FLI1* binds to the *BCL2* promoter. (A) Location of PCR primers used for *FLI1* binding *BCL2*. Two primer sets SJ4158/SJ4159 and SJ4160/SJ4161 were used to quantitate *FLI1* in promoter and exon1 of *BCL2*. The primer set SJ4154/SJ4155 (5'Ctl) was used as the negative control. (B) *FLI1* in the *BCL2* promoter of U87MG cells (Left) and U251MG cells (Right). *FLI1* in the *BCL2* promoter were measured by ChIP assay. Normal mouse IgG was used as a negative control and was used for normalization. The data are the mean ± SD from three independent experiments. ***P<0.001 as compared with control cells (CTL). ns: Not statistically significant.

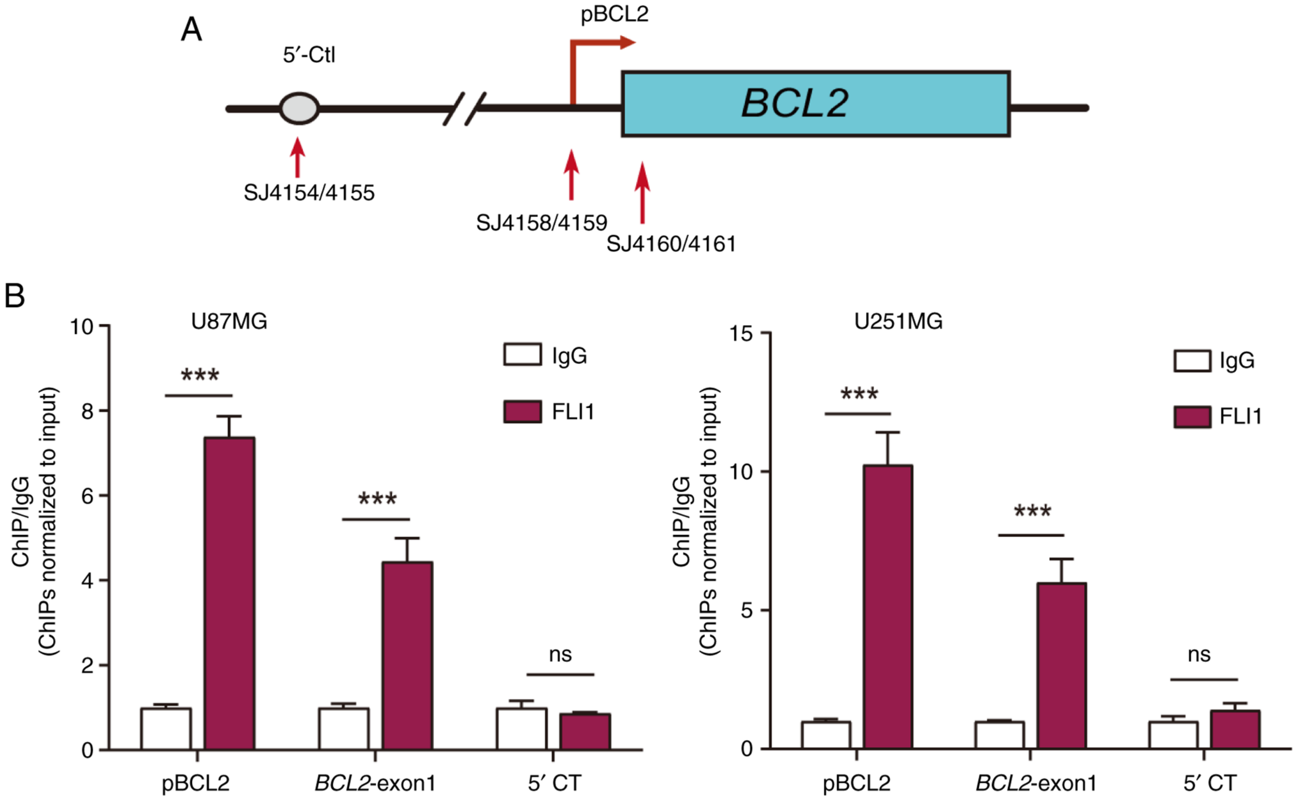

Figure S7. *FLI1* overexpression induces DNA demethylation in the *ISG15* promoter. Alteration of DNA methylation at the *ISG15* site in U87MG cells. Numbers in parenthesis: Percentage of methylated CpGs. Note the demethylation status DNA methylation in OE FLI1. OE, overexpression.

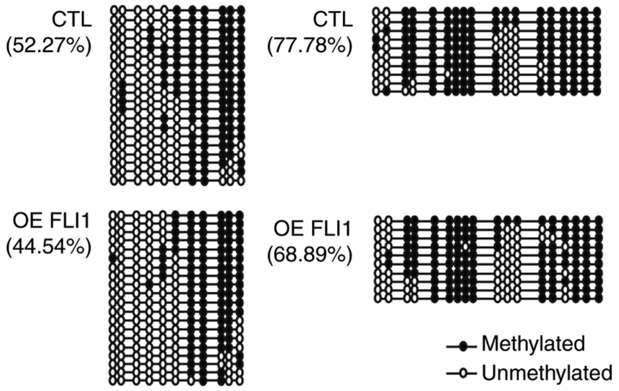

Supplement: Supplementary file 1 [file IJMM-58-3-05912-Supplementary_Data1.pdf]
