# Supplementary material for: FLI1 enhances the malignant phenotype of glioma cells and exerts immunomodulatory effects through feedback crosstalk with exonic circRNA FECR1 and interferon-induced ISG15
Source: Int J Mol Med. 2026 Jul 1;58(3):241. doi: 10.3892/ijmm.2026.5912 (PMC13354330; doi:10.3892/ijmm.2026.5912)
Supplement: Supplementary file 2 [file IJMM-58-3-05912-Supplementary_Data2.pdf]

Table SI. Oligonucleotide used for small interfering RNA.

Oligo name	Sense	Antisense
FLI1-1142	CCCUUCUGACAUCUCCUACAUTT	AUGUAGGAGAUGUCAGAAGGGTT
FLI1-294	CCCAUGAACUACAACAGCUAUTT	AUAGCUGUUGUAGUUCAUGGGTT
ISG15-142	CAUGUCGGUGUCAGAGCUGAATT	UUCAGCUCUGACACCGACAUGTT
ISG15-320	CUGAGCAUCCUGGUGAGGAAUTT	AUUCCUCACCAGGAUGCUCAGTT
Negative control	UUCUCCGAACGUGUCACGUTT	ACGUGACACGUUCGGAGAATT

Table SII. Oligonucleotide used for short hairpin RNA.

Oligo name	Targeting of the sequence
FLL1-1	CCCATGAACTACAACAGCTAT
FLL1-2	CCCTTCTGACATCTCCTACAT
ISG15-1	CATGTCGGTGTCAGAGCTGAA
ISG15-2	CTGAGCATCCTGGTGAGGAAT
Negative control	TTCTCCGAACGTGTCACGT

Table SIII. Oligonucleotide primers used for reverse transcription-PCR.

ID	Oligo name	Oligo sequence	Product size
FLL1	SJ205	AGGGGCACAAACGATCAGTA	124bp
	SJ206	GAATTGCCACAGCTGGATCT	
ISG15	SJ4002	CTCTGAGCATCCTGGTGAGGAA	136bp
	SJ4003	AAGGTCAGCCAGAACAGGTCGT	
FECR1	SJ3827	CTGTTGTCACACCTCAGTTAC	198bp
	SJ3829	CTGGCTGATTGATCCACTCCTGC	
β-actin	J880	CAGGTCATCACCATTGGCAATGAGC	135bp
	J881	CGGATGTCCACGTCACACTTCATGA	
U2	JH1055	ATCTGTTCTTATCAGTTTAATATCTG	151bp
	JH1056	GGGTGCACCGTTCCTGGAGGTAC	

Table SIV. Oligonucleotide primers used for *FECR1 CRIST assay*.

Oligo name		Oligo sequence	Product size(bp)
5'-Ctl	JH2521	GCATAATCTTATTTGTCCTCCAATAACC	124
	JH2522	GCAGAAAAGTTCTCCCACAAGC	
P1	JH4053	CTCGGTTTTCGTCCGAGTCTTC	119
	JH4054	TTTTTCCAGCCGGAGACAAACT	
P2	JH2756	GGGCTGCGAGGTCAGGCT	106
	JH4528	GTTGCCCCGCCGCTTACCTTA	
E1	JH2528	AGGGAGCTATAAGAGCCTAT	163
	JH2529	GAGACACTTGCATGAACACATC	

Table SV. Oligonucleotide primers used for *ChIP*.

A, ISG15 CHIP

Oligo name		Oligo sequence	Product size (bp)
A	SJ4150	ACACCAATCTGAGCAAATACTG	158
	SJ4151	GCTGTCATCACATGGACTAATG	
B	SJ4152	TTATAATAGGGCCGGTGCTGCC	119
	SJ4153	GCCTTACCATGGCTGTGGGCTG	
C	SJ4154	CCACTGACGGCGTCCAACTC	134
	SJ4155	CTCTCCAGAAGAGCAGAGGCAAG	

B, BCL2 CHIP

Oligo name		Oligo sequence	Product size (bp)
A	SJ4158	ACCAGGAGGAGGAGAAAGGGTG	108
	SJ4159	CTAAAAAGGATGACTGCTACGAAGTTC	
B	SJ4160	CGCGTCCTGCCTTCATTTATCCAG	169
	SJ4161	CAGGCATGAATCTCTATCCACGGG	

Table SVI. Oligonucleotide primers used for Chromosome conformation capture.

ID	Oligo name	Oligo sequence
promoter, 1st	SJ4510	ACACCAATCTGAGCAAATACTG
promoter, 2nd	SJ4260	GAGTGTTGTTATCTCTGGGTAG
promoter, 1st	SJ4261	TGCCCCAGAGTGAGCGGAAG
promoter, 2nd	SJ4262	CCGAATGACCGAAAGACAGGG
promoter, 1st	SJ4263	GTGGGCTCTGTGCCAGCC
promoter, 2nd	SJ4264	TCTCCCTCCCCCAGCCAAG
promoter, 1st	SJ4265	CCAGTGCCTTGTGTGTGG
promoter, 2nd	SJ4266	TGGCGCCGCAGTCTCTGAAC
5'enhancer 1,1st	SJ4267	CGCGGGAAGTCGGGAAGG
5'enhancer 1,2nd	SJ4268	CTCGGTCTCGAGCCTCTTGG
5'enhancer 1,1st	SJ4269	CTCTGTCGCTCCCAAGGCTTTC
5'enhancer 1,2nd	SJ4270	TGCAGCCCTCCGTGCTCCA
5'enhancer 2,1st	SJ4271	GGCCGGCCATTGTGTCTG
5'enhancer 2,2nd	SJ4272	CACCCTCCCTCCTTCCTG
5'enhancer 2,1st	SJ4273	CGGCAGCTGAGGGACAGC
5'enhancer 2,2nd	SJ4274	CCAGGTGTCCAGCATGGC
5'enhancer 2,1st	SJ4275	CTCTCTGCCAGAGCCGTG
5'enhancer 2,2nd	SJ4276	CCTGGAGTCCTACCCACTCTC
3'enhancer, 1st	SJ4277	GAGGCAGGCGGACCACTTG
3'enhancer, 2nd	SJ4278	GACCAGCCTGGCCAACATGATG
3' control	SJ4279	GTTACCGTGCCCTGTCTG
3' control	SJ4280	ATGGGCCTGGCCTAGACTTC
5' control	SJ4281	CTATAAAGAAACGCCTGACAG

Table SVII. Oligonucleotide primers used for *ISG15 DNA methylation.*

Oligo name		Oligo sequence	Product size (bp)
CpG1	SJ4193	TTAGTGTTTTGTGTGTGGTGGGTT	255
	SJ4194	CCCTAACTAACAAAAAAAACCCTATCCTA	
CpG2	SJ4195	TAGGGTTTTTTTTGTTAGTTAGGGTT	378
	SJ4196	ACCTTTATTTCCRACCCTTAATCCTA	
